# MEK inhibitor mirdametinib promotes fracture healing in osteofibrous dysplasia RASopathy

**DOI:** 10.1172/JCI199048

**Published:** 2026-02-26

**Authors:** Aysha B. Khalid, Kristin Denton, Nandina Paria, Ila Oxendine, Meghan Wassell, Reuel Cornelia, Sasidhar Uppuganti, Jeffry S. Nyman, G. Jayashree Jagadeesh, Carlos R. Ferreira, Simon J. Conway, Robert E. Hammer, John Ritter, Mylinh Nguyen, David A. Podeszwa, Laura J. Klesse, Carol A. Wise, Jonathan J. Rios

**Affiliations:** 1 Center for Translational Research, Scottish Rite for Children, Dallas, Texas, USA.; 2 Department of Orthopaedic Surgery, Vanderbilt University Medical Center, Nashville, Tennessee, USA.; 3 Department of Biomedical Engineering, Vanderbilt University, Nashville, Tennessee, USA.; 4 United States Department of Veterans Affairs, Tennessee Valley Healthcare System, Nashville, Tennessee, USA.; 5 Unit on Skeletal Genomics, Eunice Kennedy Shriver National Institute of Child Health and Human Development, NIH, Bethesda, Maryland, USA.; 6 Herman B. Wells Center for Pediatric Research, Indiana University School of Medicine, Indianapolis, Indiana, USA.; 7 Department of Biochemistry, UT Southwestern Medical Center, Dallas, Texas, USA.; 8 Department of Orthopaedics, Scottish Rite for Children, Dallas, Texas, USA.; 9 Department of Orthopaedic Surgery,; 10 Department of Pediatrics, and; 11 Simmons Comprehensive Cancer Center, UT Southwestern Medical Center, Dallas, Texas, USA.; 12 Department of Pediatrics, Children’s Health, Dallas, Texas, USA.; 13 McDermott Center for Human Growth and Development, UT Southwestern Medical Center, Dallas, Texas, USA.

**Keywords:** Bone biology, Clinical Research, Genetics, Bone disease, Drug therapy

## Abstract

Osteofibrous dysplasia (OFD) is a skeletal RASopathy presenting with periosteal bone lesions that may progress to fracture and delayed healing (pseudarthrosis). *MET* gene mutations reducing ubiquitin-mediated protein degradation via loss of the juxtamembrane domain (MET^ΔJMD^) were previously identified in patients with OFD, resulting in ligand-dependent gain of function. The effect of *MET*^ΔJMD^ expression on skeletal progenitor cell differentiation and the potential efficacy of targeted therapies remain unclear. We engineered *Met*^ΔJMD^ mice and showed that *Met*^ΔJMD^ expression inhibited osteogenic differentiation of skeletal progenitor cells in vitro and impaired cortical bone development and reduced bone stiffness in vivo. In contrast, conditional deletion of *Met* enhanced osteogenic differentiation of periosteal progenitor cells. Inhibition of MAPK signaling with MEK inhibitors restored osteogenic differentiation of mouse *Met*^ΔJMD^ skeletal progenitor cells and promoted the activation of transcriptional signatures associated with skeletal development and osteoblast differentiation in pseudarthrosis-derived primary cells from patients with OFD. With this preclinical support, we treated with the MEK inhibitor mirdametinib a pediatric patient with OFD who had a 3-year history of persistent pseudarthrosis, resulting in fracture union. Our findings demonstrate a bidirectional role for MET in regulating osteogenic differentiation of skeletal progenitor cells and a therapeutic avenue to improve clinical outcomes for this and potentially other skeletal RASopathies.

## Abstract 2

This study identifies MAPK pathway activation in the pathogenesis of osteofibrous dysplasia and demonstrates efficacy of the MEK inhibitor mirdametinib in a pediatric patient.

## Introduction

Osteofibrous dysplasia (OFD) (OMIM# 607278) is a congenital skeletal disorder affecting the long bones of children (1). OFD is defined by radiolucent lesions at the periosteal surface affecting the cortical bone. Lesions are often focal, although multifocal involvement of the same bone has been observed. OFD lesions may also be associated with bowing and pathologic fracture that may fail to heal and develop persistent pseudarthrosis. While OFD is most often sporadic and unilateral, families with inherited OFD as well as rare cases of bilateral OFD have been described (2–5). Histologic diagnosis of OFD is characterized by osteoblast rimming of woven bone with a central fibrous osteolytic nidus, which distinguishes OFD from other conditions, such as fibrous dysplasia. Some OFD lesions may resolve spontaneously; however, surgical en bloc excision may be needed for progressive lesions or for persistent fracture pseudarthrosis (6).

The MET proto-oncogene RTK protein consists of an extracellular domain, a transmembrane region, a juxtamembrane domain (JMD), and a kinase domain. Phosphorylation sites within the JMD and kinase domains negatively and positively regulate MET activity, respectively. Activation of the MET receptor requires binding of its ligand, hepatocyte growth factor (HGF), which leads to phosphorylation of kinase domain tyrosine residues, protein internalization, and activation of downstream effector pathways such as the RAS/RAF/MEK/ERK (MAPK), PIK3CA/AKT/mTOR, and STAT pathways, among others (7–11). After the initiation of this signaling cascade, the receptor is recycled back to the cell surface or undergoes ubiquitin-mediated degradation via phosphorylation of the JMD tyrosine residue (Tyr_1003_) by the CBL ubiquitin ligase.

Inherited and somatic pathogenic *MET* gene variants were previously identified in patients with OFD who had familial and sporadic disease, respectively (4). Inherited variants were localized to the exon 14 splice donor site, which resulted in exon skipping and in-frame exclusion of the JMD in an otherwise full-length protein. To date, the only somatic OFD variant detected is a point mutation at the Tyr_1003_ residue within the JMD. Splice exclusion of the JMD or mutation of the Tyr_1003_ residue leads to loss of ubiquitin-mediated degradation of the receptor and prolonged pathway activation (4, 10). Therefore, OFD-causing variants lead to a ligand-dependent gain-of-function MET receptor, as has been described in human cancers (12).

The molecular context of *MET*^ΔJMD^ expression is best demonstrated in cancer, where it promotes tumor growth via an “oncogene addiction” mechanism (13). Inhibition of the addicted pathway results in cancer cell death, and, consequently, MET inhibitors were approved to treat non–small cell lung cancer (NSCLC) associated with *MET*^ΔJMD^ expression (14, 15). However, the effect of *MET*^ΔJMD^ expression on skeletal progenitor cell differentiation or skeletal development remains unstudied, and thus no evidence yet exists regarding the use of targeted therapies to treat the OFD RASopathy.

Here, we demonstrate that Met bidirectionally regulates osteogenic differentiation, in part, via regulation of MAPK signaling. Inhibition of MAPK signaling with MEK inhibitors rescued osteogenic differentiation of skeletal progenitor cells from *Met*^ΔJMD^ mice and promoted the expression of osteogenic gene signatures in OFD patient pseudarthrosis–derived primary cells. Finally, we present the successful treatment of a long-term, persistent pseudarthrosis in a patient with OFD with the MEK inhibitor mirdametinib. Results from our study support the potential use of MEK inhibitors to treat pseudarthrosis associated with OFD and, potentially, other RASopathies.

## Results

### Met^ΔJMD^ expression promotes ERK pathway activation in skeletal progenitor cells.

**Figure 1:**
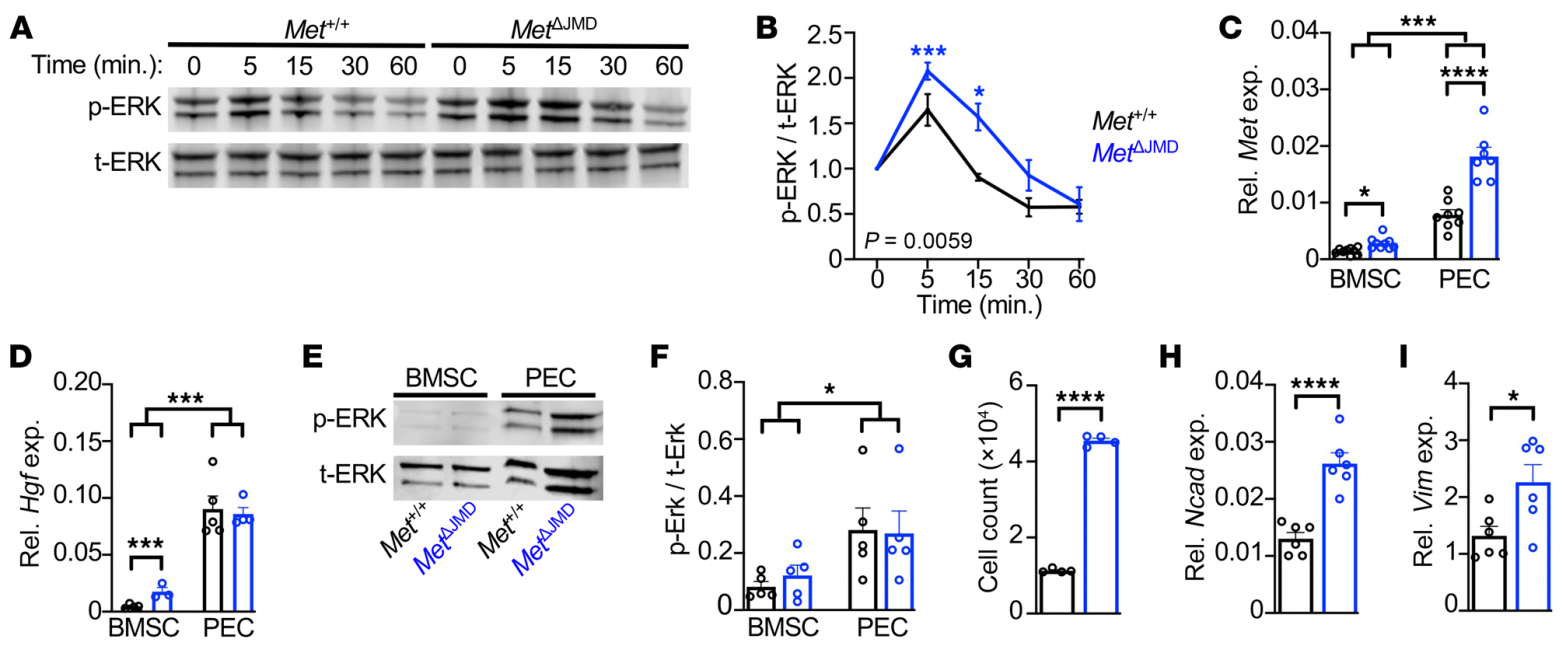
Characterization of *Met*^ΔJMD^ osteoprogenitor cells. (**A** and **B**) Representative Western blot time course (**A**) and quantification (**B**) of ERK activation following HGF stimulation of BMSCs from control (black; *n* = 3) and *Met*^ΔJMD^ (blue; *n* = 5) mice. Statistical analysis was conducted by 2-way ANOVA with Dunnett’s multiple-test correction including only 5- and 15-minute time points. (**C** and **D**) Relative (Rel.) expression (exp.) of *Met* (**C**) and *Hgf* (**D**) in BMSCs and PECs from control (*n* = 8–9) and *Met*^ΔJMD^ (*n* = 7–9) mice. Statistically significant differences were determined by 2-way ANOVA with Šidák’s multiple-test correction. (**E** and **F**) Representative Western blot (**E**) and quantification (**F**) of ERK pathway activation of serum-starved BMSCs and PECs from control (*n* = 5) and *Met*^ΔJMD^ (*n* = 5) mice. Statistically significant differences were determined by 2-way ANOVA with Šidák’s multiple-test correction. (**G**) Cell count quantification of PECs from control (*n* = 4) and *Met*^ΔJMD^ (*n* = 4) mice. Statistically significant differences were determined by *t* test. (**H** and **I**) Relative expression of the mesenchymal adhesion genes *Ncad* (**H**) and *Vim* (**I**) in PECs from control (*n* = 6) and *Met*^ΔJMD^ (*n* = 6) mice. (**G**–**I**) Statistically significant differences were determined by 2-tailed *t* test. RT-qPCR values from cultured cells and other quantification data are presented as the mean ± SEM. *P < 0.05, ***P < 0.001, and ****P < 0.0001.

To investigate the effect of *Met*^ΔJMD^ expression on skeletal progenitor cell differentiation and skeletal development, we deleted the JMD-encoding *Met* exon 15 in mice using CRISPR/Cas9 (Supplemental Figure 1A; supplemental material available online with this article; https://doi.org/10.1172/JCI199048DS1). Heterozygous *Met*^+/ΔJMD^ mice (herein referred to as *Met*^ΔJMD^) were viable and fertile with no overt phenotype. Similar to mice harboring constitutive activating *Met* kinase domain variants (16), homozygous *Met*^ΔJMD/ΔJMD^ mice were embryonically lethal. Ligand-dependent gain of function due to loss of the MET JMD in lung cancer cell lines results in prolonged MAPK pathway activation following stimulation with HGF (17). Therefore, we isolated bone marrow stromal cells (BMSCs) from adult control and *Met*^ΔJMD^ mice, treated the cells with HGF, and quantified relative ERK activation and normalization. In both control and *Met*^ΔJMD^ BMSCs, the ERK pathway was quickly activated in response to HGF, although normalization following stimulation was significantly delayed in *Met*^ΔJMD^ BMSCs compared with the control (Figure 1, A and B). Thus, *Met*^ΔJMD^ mice harbor a heterozygous germline deletion of the endogenous *Met* JMD-encoding exon leading to ligand-dependent gain of function in MAPK signaling.

OFD lesions occur at the periosteal surface of bone, and *Met* expression was previously detected at the periosteal surface of mouse embryonic long bones (4). Therefore, we cultured leptin receptor–expressing (*LepR*-expressing) BMSCs and periostin-expressing (*Postn*-expressing) periosteal explant cells (PECs) from control and *Met*^ΔJMD^ mice (Supplemental Figure 1, B and C) and tested expression of *Met* and *Hgf* in both cell types. *Met* expression was significantly higher in PECs compared with BMSCs (*P* < 0.0001). Compared with control cells, we found that *Met* expression was higher in *Met*^ΔJMD^ BMSCs and PECs (Figure 1C). This difference was predominantly due to the relative overexpression of the *Met*^ΔJMD^ allele (Supplemental Figure 1, D and E), implicating, for the first time to our knowledge, an allele-specific feedback mechanism associated with the *Met*^ΔJMD^ allele. While *Hgf* expression was modestly elevated in *Met*^ΔJMD^ BMSCs compared with the control, *Hgf* expression was significantly higher in PECs from both control and *Met*^ΔJMD^ mice (Figure 1D). Consistent with the higher *Met* and *Hgf* expression in PECs, ERK activation was significantly higher in serum-starved control and *Met*^ΔJMD^ PECs compared with BMSCs (Figure 1, E and F). We observed no differences in AKT/mTOR pathway activation, as assayed by phosphorylated S6 (p-S6) (Supplemental Figure 1F). These results implicate a molecular basis for the periosteal localization of OFD lesions observed in patients.

Last, MET activation enhances tumor growth and invasion, in part, through promotion of epithelial-mesenchymal transition (18–21). Therefore, we tested cell adhesion and the expression of mesenchymal cell adhesion–associated genes in control and *Met*^ΔJMD^ PECs. Compared with control cells, *Met*^ΔJMD^ PECs demonstrated significantly greater adherence in culture concomitant with significantly higher expression of the cell adhesion genes N-cadherin (*Ncad*) and vimentin (*Vim*) (Figure 1, G–I), although no difference in cell proliferation was observed (Supplemental Figure 1, G and H). Taken together, these results demonstrated molecular and cellular defects in skeletal progenitor cells from *Met*^ΔJMD^ mice associated with ERK pathway activation.

### Impaired progenitor cell differentiation and skeletal development in Met^ΔJMD^ mice.

**Figure 2:**
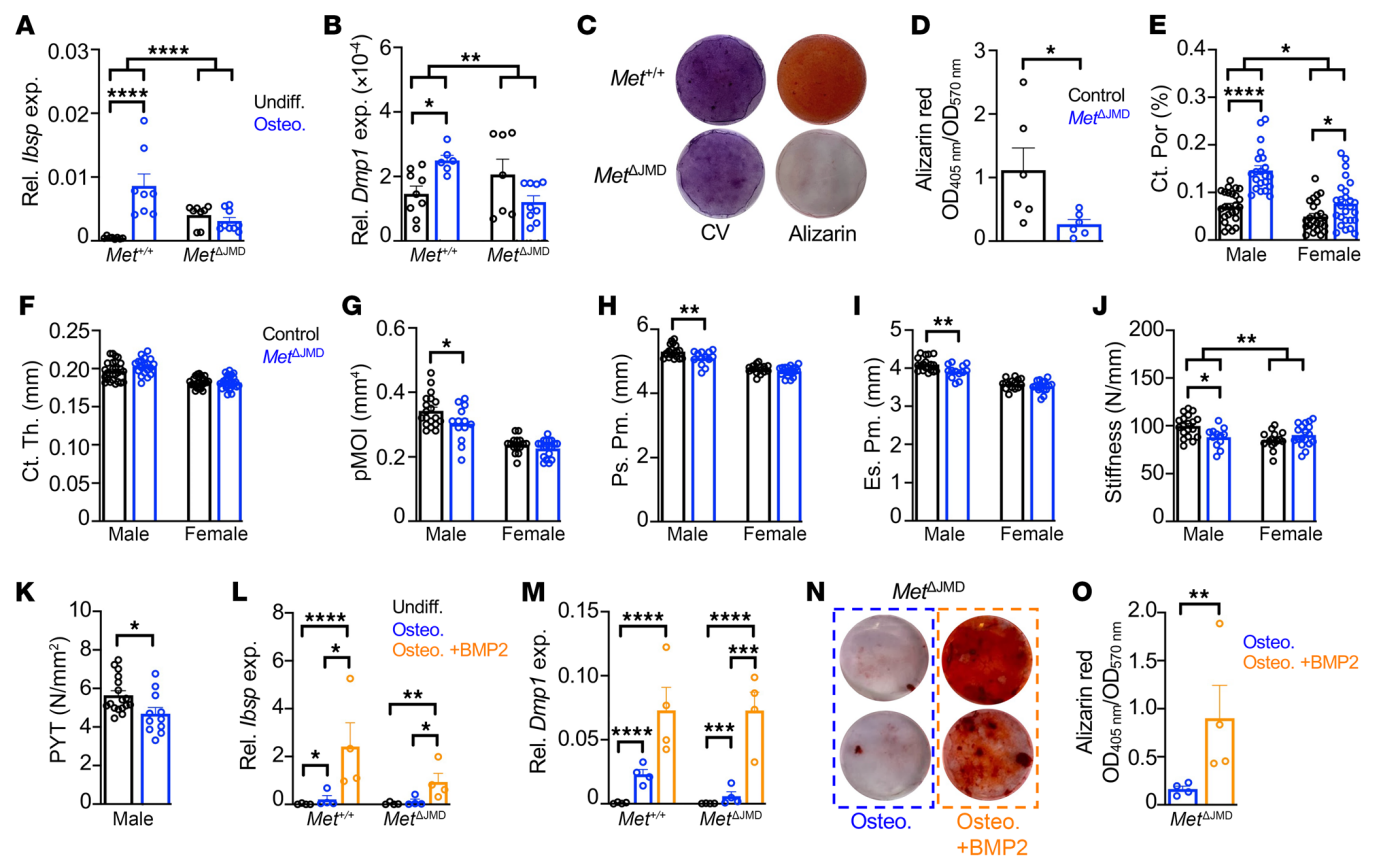
Impaired osteogenic differentiation of PECs from *Met*^ΔJMD^ mice. (**A** and **B**) Relative expression of the osteogenic genes *Ibsp* (**A**) and *Dmp1* (**B**) in control (*n* = 4–9) and *Met*^ΔJMD^ (*n* = 4–9) PECs prior to (undifferentiated [Undiff.], black) and following osteogenic differentiation (Osteo., blue). Statistically significant differences were determined by 2-way ANOVA with Šidák’s multiple-test correction. (**C** and **D**) Representative alizarin red and crystal violet (CV) staining (**C**) and quantification (**D**) following osteogenic differentiation of control (Met^+/+^, *n* = 6) and *Met*^ΔJMD^ (*n* = 6) PECs. Statistically significant differences were determined by *t* test. (**E**–**J**) Quantification of cortical porosity (**E**) (Ct. Por.), cortical thickness (**F**) (Ct. Th.), pMOI (**G**), periosteal perimeter (**H**) (Ps. Pm), endosteal perimeter (**I**) (Es. Pm.), and stiffness (**J**) of femurs from 4-month-old male and female control (black; *n* = 15–49) and *Met*^ΔJMD^ (blue; *n* = 13–47) male and female mice. Statistically significant differences were determined by 2-way ANOVA with Šidák’s multiple-test correction. (**K**) Quantification of PYT of femurs from 4-month-old male control (*n* = 17) and *Met*^ΔJMD^ (*n* = 11) mice. Statistically significant differences were determined by *t* test. (**L** and **M**) Relative expression of the osteogenic genes *Ibsp* (**L**) and *Dmp1* (**M**) prior to differentiation (Undiff., black) or following standard osteogenic differentiation (Osteo., blue) or osteogenic differentiation with BMP2 (Osteo.+BMP2, orange) in control (*n* = 5) and *Met*^ΔJMD^ (*n* = 5–6) PECs. Statistically significant differences were determined by 2-way ANOVA with Tukey’s multiple-test correction. (**N** and **O**) Representative alizarin red staining (**N**) and quantification (**O**) following osteogenic differentiation of *Met*^ΔJMD^ PECs. (**D**, **K**, and **N**) Statistically significant differences were determined by 2-tailed *t* test (*n* = 4 per group). RT-qPCR from cultured cells and other quantification data are presented as the mean ± SEM. *P < 0.05, **P < 0.01, ***P < 0.001, and ****P < 0.0001.

In osteoprogenitor cells, MAPK homeostasis is essential for proper osteogenic differentiation and mineralization. Preclinical cellular and rodent models of skeletal RASopathies, such as in neurofibromatosis type 1 (NF1) (22, 23) and others (24), have implicated impaired osteogenic differentiation and/or mineralization in the pathogenesis of disease. Therefore, we tested osteogenic differentiation of *Met*^ΔJMD^ PECs by measuring the relative expression of early and late osteogenic genes following differentiation, including integrin-binding sialoprotein (*Ibsp*), RUNX family transcription factor 2 (*Runx2*), osterix (*Osx*), osteocalcin (*Ocn*), and dentin matrix acidic phosphoprotein 1 (*Dmp1*). While expression of all osteogenic genes was significantly increased in control PECs following differentiation, expression of these genes was mostly unchanged in *Met*^ΔJMD^ PECs, suggesting impaired osteogenic differentiation in *Met*^ΔJMD^ PECs (Figure 2, A and B, and Supplemental Figure 2, A–C). Consistent with this, mineralization measured by alizarin red staining was significantly reduced following differentiation of *Met*^ΔJMD^ PECs compared with the control (Figure 2, C and D). These results demonstrate that expression of the *Met*^ΔJMD^ allele impaired osteogenic differentiation and mineralization of periosteal osteoprogenitor cells, potentially due to MAPK activation.

To evaluate whether in vitro defects in osteoblast differentiation and mineralization correlate with skeletal deficiencies in vivo, we analyzed the microstructure and biomechanical properties of the long bones from adult control and *Met*^ΔJMD^ mice. Trabecular bone microarchitecture was unchanged in *Met*^ΔJMD^ mice compared with the control (Supplemental Figure 2, D–G), and we observed no differences in surface osteoblasts or osteoclasts between control and *Met*^ΔJMD^ mice (Supplemental Figure 2, H and I). In contrast, cortical porosity, but not cortical thickness, was significantly increased at the femur mid-diaphysis in *Met*^ΔJMD^ mice, with greater differences observed in male versus female mice (Figure 2, E and F). The polar moment of inertia (pMOI) of the femur mid-diaphyseal cortex, a structural parameter sensitive to the periosteal circumference, was lower in 4-month-old male *Met*^ΔJMD^ mice (Figure 2G). Likewise, both the periosteal and endosteal circumferences were reduced in male *Met*^ΔJMD^ mice (Figure 2, H and I). Consistent with increased cortical porosity, biomechanical testing further demonstrated reduced stiffness and post-yield toughness (PYT) in the femurs of male *Met*^ΔJMD^ mice compared with controls (Figure 2, J and K). Taken together, *Met*^ΔJMD^ expression inhibited osteogenic differentiation of periosteal progenitor cells, resulting in impaired cortical bone development in mice, leading to decreased resistance to an applied load.

OFD lesions may progress to fracture, which is often treated with recombinant bone morphogenetic protein 2 (BMP2) applied to the outer periosteal bone surface. Therefore, we tested whether exogenous BMP2 can rescue osteogenic differentiation defects inherent in *Met*^ΔJMD^ PECs. Osteogenic gene expression was significantly increased following osteogenic differentiation with BMP2 of both control and *Met*^ΔJMD^ PECs (Figure 2, L and M, and Supplemental Figure 2, J–L). Alizarin red staining confirmed the rescue of osteoblast mineralization following differentiation of *Met*^ΔJMD^ PECs with BMP2 (Figure 2, N and O). These results demonstrate that *Met*^ΔJMD^-expressing skeletal progenitor cells remained responsive to the anabolic effects of BMP2 despite activated MAPK signaling. The intact responsiveness to BMP2 is consistent with the lack of overt skeletal deformity in *Met*^ΔJMD^ mice.

### Reduced Met expression enhances osteogenic differentiation of PECs.

**Figure 3:**
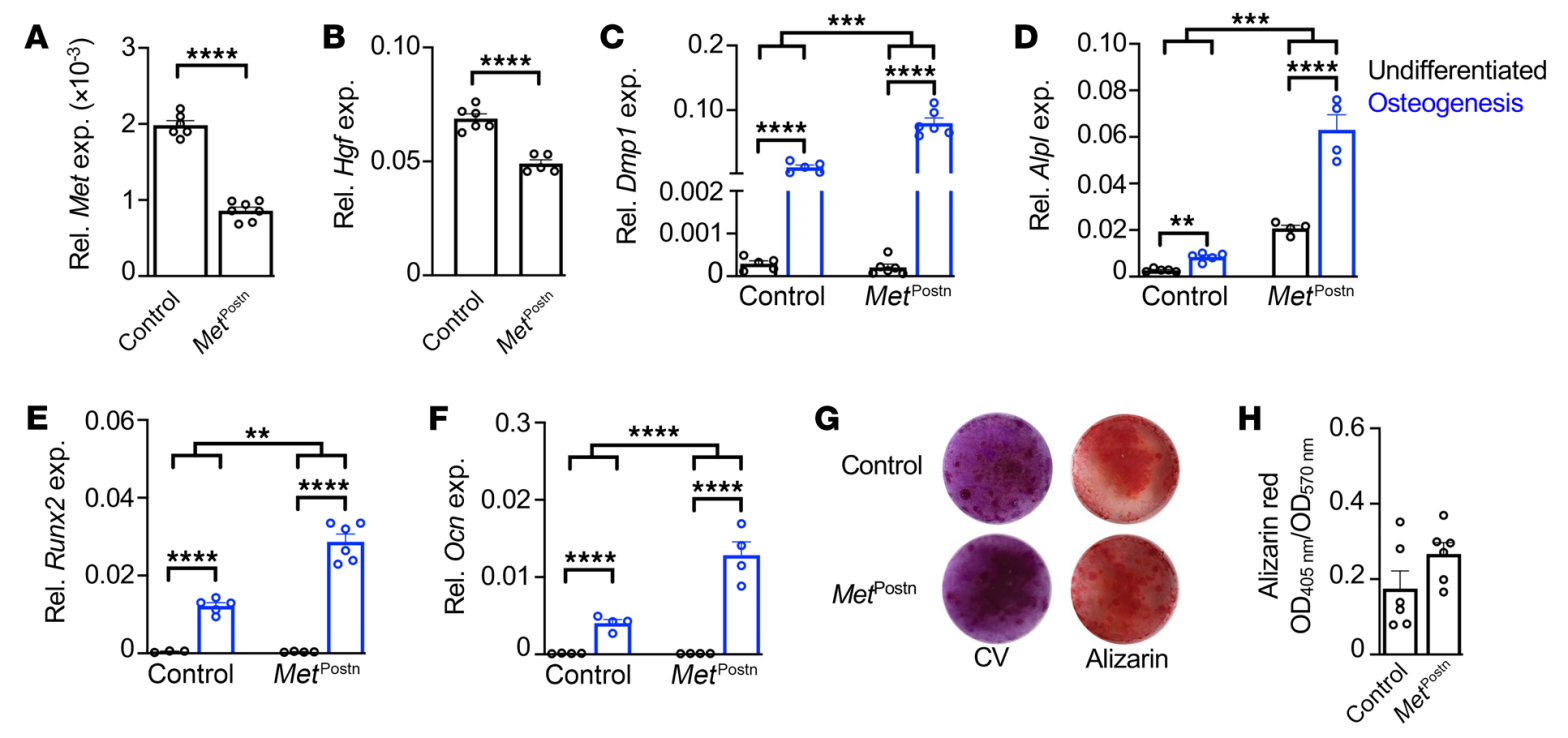
Osteogenic differentiation of PECs from *Met*^Postn^ mice. (**A** and **B**) Relative expression of *Met* (**A**) and *Hgf* (**B**) in PECs from control (*n* = 6) and *Met*^Postn^ (*n* = 7) mice. Statistically significant differences were determined by *t* test. (**C**–**F**) Relative expression of the osteogenic marker genes *Dmp1* (**C**), *Alpl* (**D**), *Runx2* (**E**), and *Ocn* (**F**) prior to (Undiff., black) and following osteogenic differentiation (Osteogenesis, blue) of PECs from control (*n* = 3) and *Met*^Postn^ (*n* = 4–6) mice. Statistically significant differences were determined by 2-way ANOVA with Šidák’s multiple-test correction. (**G** and **H**) Representative crystal violet and alizarin red staining (**G**) and quantification (**H**) of PECs from control and *Met*^Postn^ mice following osteogenic differentiation (*n* = 6 per group). **A**–**C**, **H**, and **K**) Statistically significant differences were determined by *t* test. RT-qPCR from cultured cells and other quantification data are presented as the mean ± SEM. **P < 0.01, ***P < 0.001, and ****P < 0.0001.

*Met*^ΔJMD^ expression significantly inhibited osteogenic differentiation of PECs; therefore, we next tested whether loss of *Met* promotes PEC osteogenesis. To test this, we cultured PECs following conditional deletion of *Met* in the bone periosteum of *Postn*-Cre *Met^fl/fl^* mice (herein referred to as *Met*^Postn^). Expression of *Met*, and to a lesser extent *Hgf*, was significantly reduced in *Met*^Postn^ PECs compared with the control (Figure 3, A and B). To test osteogenic differentiation, we measured the expression of the osteogenic genes *Dmp1* and *Alpl* in control and *Met*^Postn^ PECs. Following differentiation, the expression of osteogenic genes was significantly higher in *Met*^Postn^ PECs than in differentiated control PECs (Figure 3, C–F). Likewise, there was suggestive evidence (*P* = 0.08) for increased mineralization following differentiation of *Met*^Postn^ PECs compared with control (Figure 3, G and H). Taken together with results from *Met*^ΔJMD^ PECs, these results suggest that Met regulates osteogenic differentiation of cultured PECs and that inhibition of Met, or downstream MAPK signaling, may rescue osteogenic differentiation of *Met*^ΔJMD^ PECs.

### MEK inhibition rescues differentiation and mineralization of Met^ΔJMD^ PECs.

**Figure 4:**
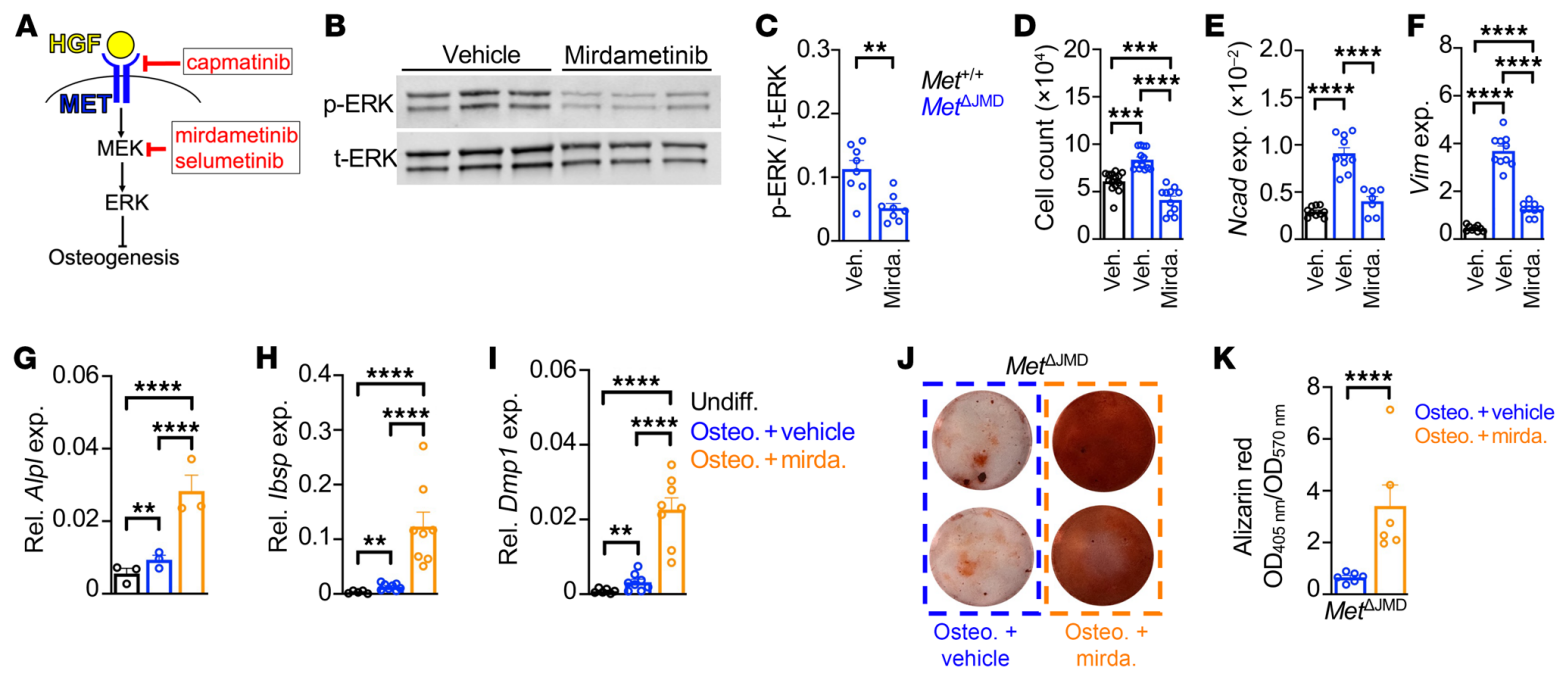
MEK inhibition rescues osteogenic differentiation of *Met*^ΔJMD^ PECs. (**A**) Schematic of the MET receptor (blue) bound by HGF (yellow) to activate the downstream MEK/ERK signaling cascade, resulting in the regulation of osteogenic differentiation of skeletal progenitor cells. Pharmacologic inhibitors of MET (capmatinib) and MEK (mirdametinib, selumetinib) are shown in red. (**B** and **C**) Representative Western blot (**B**) and quantification (**C**) of ERK pathway activation in *Met*^ΔJMD^ PECs treated with vehicle (*n* = 8) or mirdametinib (mirda.) (*n* = 8). Statistically significant differences were determined by *t* test. (**D**) Cell counts of PECs from control (*n* = 14) and *Met*^ΔJMD^ (*n* = 14) mice. Statistically significant differences were determined by 1-way ANOVA with Tukey’s multiple-test correction. (**E** and **F**) Relative expression of the cell adhesion genes *Ncad* (**E**) and *Vim* (**F**) in control (*Met*^+/+^; *n* = 9–10) and *Met*^ΔJMD^ (*n* = 7–10) PECs treated with vehicle (Veh.) or mirdametinib. Statistically significant differences were determined by 1-way ANOVA with Tukey’s multiple-test correction. (**G**–**I**) Relative expression of the osteogenic genes *Alpl* (**G**), *Ibsp* (**H**), and *Dmp1* (**I**) in *Met*^ΔJMD^ PECs prior to (Undiff., black) or following osteogenic differentiation in the presence of vehicle (Osteo.+vehicle, blue) or mirdametinib (Osteo.+mirda., orange) (*n* = 3–8 per group). Statistically significant differences were determined by 1-way ANOVA with Tukey’s multiple-test correction. (**J** and **K**) Representative alizarin red staining (**J**) and quantification (**K**) of *Met*^ΔJMD^ PECs following osteogenic differentiation in the presence of vehicle (blue, *n* = 6) or mirdametinib (orange, *n* = 6). Statistically significant differences were determined by *t* test. RT-qPCR from cultured cells and other quantification data are presented as the mean ± SEM. **P < 0.01, ***P < 0.001, and ****P < 0.0001.

The elevated MAPK activation associated with *Met*^ΔJMD^ expression implicates the use of targeted therapies, such as the MET inhibitor (METi) capmatinib or the MEK inhibitors (MEKi) mirdametinib and selumetinib to treat OFD (Figure 4A). Each compound was tested for its ability to rescue the cell adhesion and osteogenic differentiation defects inherent in *Met*^ΔJMD^ PECs. Capmatinib is approved by the FDA to treat lung cancers expressing the *MET*^ΔJMD^ allele or those with a high *MET* copy number (copy number >10), both of which convey the MET oncogene addiction (14, 25). In contrast, MET inhibition was ineffective in treating lung cancer with low-level *MET* amplification (copy number <10) (14). These results suggest that the efficacy of METi is context dependent, and the effectiveness of MET inhibition in OFD remains untested. As expected, cell adhesion of vehicle-treated *Met*^ΔJMD^ PECs was significantly higher compared with vehicle-treated control cells, while capmatinib treatment significantly reduced cell adhesion of *Met*^ΔJMD^ PECs to control levels (Supplemental Figure 3A). Likewise, expression of the cell adhesion genes *Ncad* and *Vim* was significantly increased in vehicle-treated *Met*^ΔJMD^ PECs but normalized to control levels with capmatinib treatment (Supplemental Figure 3, B and C). To test mineralization, control and *Met*^ΔJMD^ PECs were treated with capmatinib throughout in vitro osteogenic differentiation. Following osteogenic differentiation, capmatinib failed to rescue mineralization of *Met*^ΔJMD^ PECs (Supplemental Figure 3, D and E). These results suggest that, unlike in MET-addicted lung cancer, the expression of *Met*^ΔJMD^ in PECs is not sufficient to permit therapeutic rescue with a METi. In support of this, the level of *Met* expression in *Met*^ΔJMD^ PECs was 5 times lower than in METi-sensitive sarcomas expressing the *Met*^ΔJMD^ allele (our unpublished observations).

We next tested whether directly targeting MAPK pathway activation with the MEKi mirdametinib rescues osteogenic differentiation and mineralization of *Met*^ΔJMD^ PECs. Mirdametinib (PD0325901) is a non-ATP-competitive and selective small-molecule inhibitor of both MEK1 and MEK2 phosphorylation (26). In adults and children with NF1, mirdametinib reduced plexiform neurofibroma (pNF) tumor volume, leading to its recent approval by the FDA and the European Medicines Agency (27). To confirm the molecular response of *Met*^ΔJMD^ PECs to mirdametinib treatment, we compared MAPK pathway activation of treated cells with vehicle-treated *Met*^ΔJMD^ PECs. Compared with vehicle treatment, mirdametinib significantly reduced ERK pathway activation in *Met*^ΔJMD^ PECs (Figure 4, B and C). Mirdametinib rescued cell adhesion of treated *Met*^ΔJMD^ PECs, which was further confirmed with the significant reduction in expression of *Ncad* and *Vim* (Figure 4, D–F). We next tested the rescue of osteogenic differentiation and mineralization with mirdametinib treatment. Compared with vehicle, mirdametinib treatment significantly rescued osteogenic differentiation of *Met*^ΔJMD^ PECs, as evidenced by increases in the expression of osteogenic genes (Figure 4, G–I, and Supplemental Figure 4, A–C). Consistent with the rescue in differentiation, mirdametinib also significantly rescued mineralization in differentiated *Met*^ΔJMD^ PECs (Figure 4, J and K).

To ensure that the rescue of cell adhesion, osteogenic differentiation, and mineralization of *Met*^ΔJMD^ PECs was indeed associated with MAPK pathway inhibition, we independently repeated all experiments with selumetinib, another MEKi approved by the FDA to treat pNF in children with NF1 (28). As expected, we found that selumetinib significantly reduced ERK pathway activation in *Met*^ΔJMD^ PECs (Supplemental Figure 5, A and B) and rescued the expression of the cell adhesion genes *Ncad* and *Vim* (Supplemental Figure 5, C and D). Compared with vehicle-treated cells, selumetinib treatment throughout differentiation resulted in significantly increased the expression of osteogenic genes, consistent with rescue of osteogenesis (Supplemental Figure 5, E–J). Finally, alizarin red staining demonstrated significant rescue of mineralization of selumetinib-treated *Met*^ΔJMD^ PECs (Supplemental Figure 5, K and L). These results show that inhibition of MEK pathway activation rescued cellular and osteogenic defects inherent in *Met*^ΔJMD^ PECs and further suggest the therapeutic potential of MEKi in the treatment of children with OFD.

### MEKi enhance the expression of skeletal gene signatures in OFD patient–derived primary cells.

We previously identified a somatic p.(Tyr1003Ser) variant in primary cells cultured from a patient’s OFD fracture lesion, implicating somatic *MET* mutations in the pathogenesis of sporadic OFD–associated pseudarthrosis (4). To further evaluate somatic *MET* mutations as a cause of OFD fracture pseudarthrosis, we tested *MET*^ΔJMD^ expression by reverse transcription PCR (RT-PCR) and sequenced the JMD-encoding exon for Tyr_1003_ mutations in primary cells cultured from pseudarthroses of 4 patients with OFD. RT-PCR detected an alternatively spliced *MET* transcript in lesion-derived primary cells from 1 patient (Supplemental Figure 6A). No alternative splicing or MET JMD or kinase domain mutations were detected in the remaining 3 patient samples, possibly due to somatic mosaicism below the level of detection or to mutations present in other genes not previously associated with OFD. The alternatively spliced variant was cloned and confirmed by Sanger sequencing as the *MET*^ΔJMD^ allele (Supplemental Figure 6B). Sequencing of DNA from lesion-derived primary cells identified a somatic c.3028+2T>C splice donor variant that was not detected in DNA from a blood sample from the patient (Supplemental Figure 6C). The same allele was previously reported as a somatic mutation in lung adenocarcinomas (29, 30). We performed droplet digital PCR (ddPCR) to confirm and quantify the mutation using DNA from primary lesion cells and blood (control). The mutation burden (variant allele fraction) was 19% in the lesion-derived cells but was undetected in the blood sample (Supplemental Figure 6D), further confirming the somatic occurrence of this variant resulting in expression of the *MET*^ΔJMD^ allele. These results independently confirm that somatic *MET* mutations of the JMD domain are a cause of sporadic OFD and raise the possibility that mutations in other genes may also contribute to OFD.

To begin investigating the therapeutic potential of METi or MEKi for the treatment of OFD, we tested the effect of METi or MEKi monotherapy on OFD lesion–derived primary cells, including those from patients with and without detectable *MET* mutations. We first tested the molecular effect of the METi capmatinib on patient lesion–derived primary cells. We found that apmatinib treatment failed to significantly inhibit ERK pathway activation, and RNA-seq analysis demonstrated a minimal molecular response to METi, with few genes being differentially expressed compared with vehicle treatment (Supplemental Figure 7, A–C, and Supplemental Table 1). As with the *Met*^ΔJMD^ PECs, these results do not support a therapeutic potential for METi in OFD.

**Figure 5:**
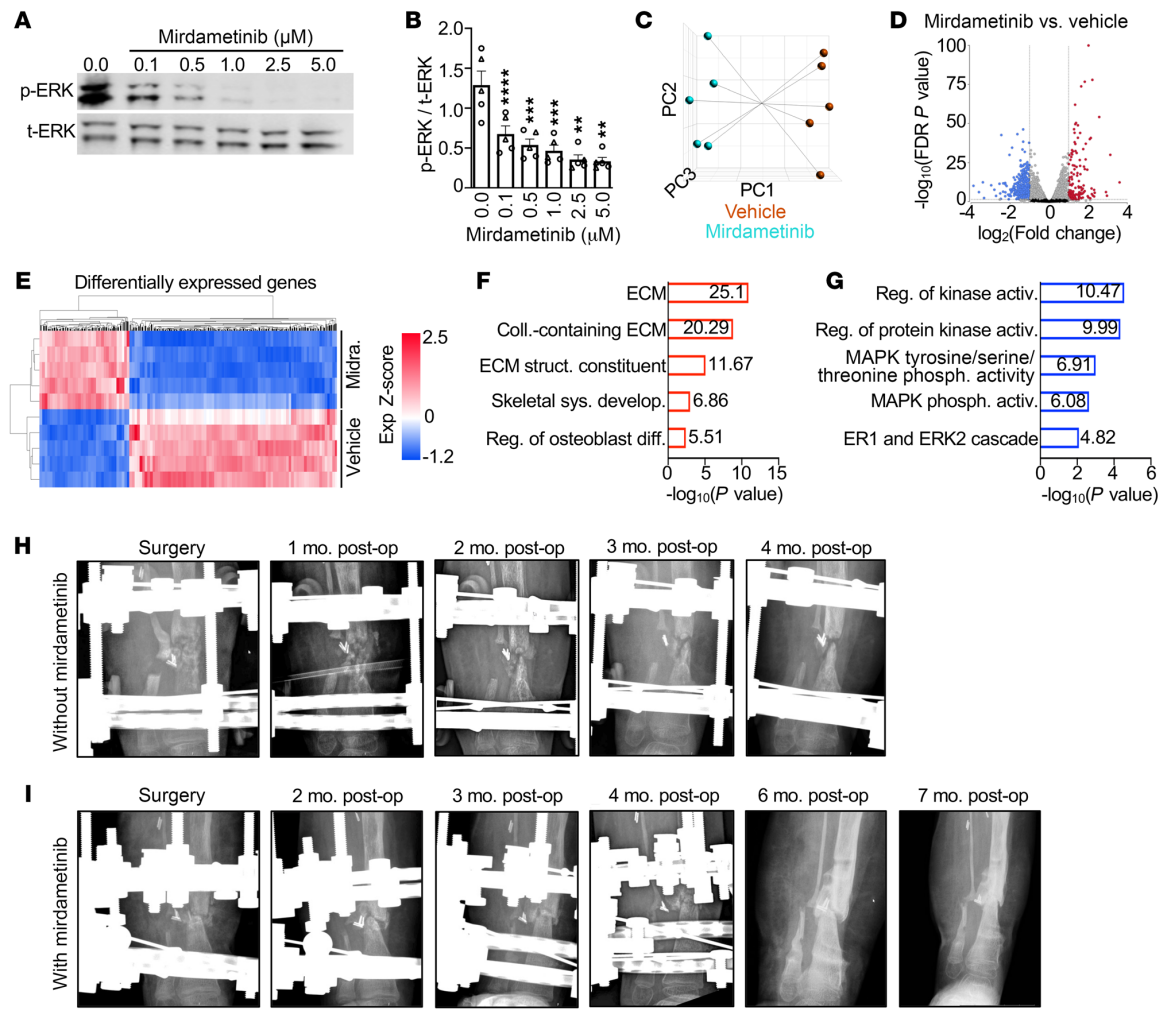
Mirdametinib improves pseudarthrosis healing in a patient with OFD. (**A** and **B**) Representative Western blot (**A**) and quantification (**B**) demonstrating a dose-dependent reduction in ERK pathway activation in OFD patient pseudarthrosis–derived primary cells (*n* = 5) treated with mirdametinib. Triangles indicate samples with somatic *MET* mutations; circles indicate samples without detectable *MET* mutations. Quantification data are presented as the mean ± SEM, with statistically significant differences determined by 1-way ANOVA with Dunnett’s multiple-test correction. (**C**) Principal component (PC) analysis of OFD patient pseudarthrosis–derived primary cells treated with vehicle or mirdametinib (*n* = 5). (**D** and **E**) Volcano plot (**D**) and heatmap (**E**) of genes differentially expressed between mirdametinib- and vehicle-treated OFD patient pseudarthrosis–derived primary cells. Exp., expression. (**F** and **G**) GO analysis of differentially expressed genes with increased (**F**) and decreased (**G**) expression following mirdametinib treatment. activ., activity; coll., collagen; diff., differentiation; phosph., phosphotase; reg., regulation; struct., structure; sys. develop., system development. (**H** and **I**) Longitudinal postoperative radiographs of an OFD patient’s pseudarthrosis treated without mirdametinib (**H**) and with mirdametinib (**I**). **P < 0.01, ***P < 0.001, and ****P < 0.0001.

We next tested the response of OFD patient lesion–derived primary cells to MEKi, including cells from patients with and without detectable *MET* gene variants. Mirdametinib significantly, and in a dose-dependent manner, inhibited ERK pathway activation (Figure 5, A and B). RNA-seq profiling distinguished vehicle-treated from mirdametinib-treated samples, identifying 333 transcripts with reduced expression and 152 transcripts with increased expression following mirdametinib treatment (Figure 5, C–E, and Supplemental Table 2). We performed gene ontology (GO) analysis to summarize the biologic response of mirdametinib in OFD lesion–derived primary cells. GO analysis results demonstrated that genes with increased expression following mirdametinib treatment were significantly associated with biologic processes required for skeletal development and fracture healing (Figure 5F and Supplemental Table 3). Genes with reduced expression following treatment were significantly enriched in MAPK pathway processes as well as biologic processes expected to be affected by MAPK pathway inhibition, such as cell-cycle regulation and cell proliferation (Figure 5G and Supplemental Table 4). These results are similar to those of a recent study of NF1 patient pseudarthrosis–derived primary cells treated with the MEKi selumetinib, which demonstrated that the molecular response to treatment was specific to MAPK hyperactive primary lesion cells, whereas patient-matched iliac crest control primary cells showed a minimal response to treatment (31).

To further demonstrate the molecular response of OFD lesion–derived primary cells to ERK pathway inhibition, we similarly tested the effects of selumetinib. ERK pathway activation was significantly reduced with selumetinib treatment (Supplemental Figure 7A). Transcriptome profiling distinguished selumetinib-treated cells from vehicle-treated cells and identified 581 transcripts with reduced expression and 331 transcripts with increased expression following selumetinib treatment (Supplemental Figure 7, B, D, and E, and Supplemental Table 5). Similar to mirdametinib, genes with increased expression following selumetinib treatment were associated with extracellular matrix formation and osteoblast differentiation, whereas genes with reduced expression were associated with MAPK pathway and kinase activity processes (Supplemental Figure 7, F and G, and Supplemental Tables 6 and 7). For all treatments, no differences were observed between patients with (*n* = 2) or without (*n* = 3) detectable *MET* mutations. Taken together, these results provide molecular evidence implicating MEKi as a potential therapeutic strategy to promote bone healing in patients with OFD.

### Mirdametinib promotes fracture healing in a patient with OFD.

Results utilizing *Met*^ΔJMD^ PECs and primary cells from patients with OFD suggested that MEKi may provide therapeutic benefit for children with OFD-associated pseudarthrosis. MEKi have become more widely utilized in the pediatric population for several indications in which the MAPK pathway is hyperactivated and have demonstrated safety and tolerability. Two MEKi resulted in inhibition of ERK pathway activation in primary cells from patients with OFD and increased the expression of genes and pathways known to promote bone formation and/or fracture healing. Therefore, we hypothesized that monotherapy with a MEKi, together with standard-of-care surgical treatment, may promote healing of OFD-associated pseudarthrosis.

Given the strong preclinical data, we administered the MEKi mirdametinib to a patient with OFD who, after multiple attempts at surgical correction, had a persistent pseudarthrosis. The patient initially presented at 1 year of age with a tibia lesion requiring resection, which resulted in persistent pseudarthrosis. The patient underwent 6 subsequent surgeries over the next 3 years due to persistent nonhealing. Multiple techniques were utilized to attempt to achieve bone union, including resection, external fixation, iliac crest grafting, and application of BMP2, although each revision failed to achieve durable union, and the patient’s pseudarthrosis persisted (Figure 5H). Local bone infection was assessed for and excluded as a cause of nonunion. A biopsy confirmed the OFD diagnosis, although no *MET* gene mutation was identified in cultured lesion-derived primary cells from the patient. Given the molecular response of patient pseudarthrosis–derived primary cells to MEKi in vitro, we sought approval to treat the patient with a MEKi. After appropriate regulatory review and approval from the SpringWorks Therapeutics Compassionate Use Program, the FDA, and the IRB at UT Southwestern Medical Center, the family consented to the use of mirdametinib. Twelve days after revision surgery that again included resection, external fixation, iliac crest grafting, and BMP2, the patient was started on mirdametinib at a dose of 1 mg twice daily via the dispersible tablet with the standard schedule of 21 days on the drug and 7 days off per a 28-day cycle. Prior to dosing, the patient underwent baseline ophthalmology evaluation, an echocardiogram, physical examination, and laboratory evaluation. The patient has been monitored on-therapy with follow-up evaluations including physical, laboratory, and cardiac evaluations. The patient has tolerated the therapy with no significant toxicity and has only noted grade 1 diarrhea in the first month of therapy that resolved by the second month of therapy. The patient has now completed 7 months of therapy with no further toxicity noted.

Fracture healing was monitored by monthly radiographs, which demonstrated progressive maturation of the iliac crest graft that, at 6 months after surgery, resulted in a durable bony bridge and union of the fracture despite early postsurgical distal segment translation leaving a residual angular deformity (Figure 5I). The external fixator frame was removed and the patient was transitioned to a long leg cast to promote further healing and remodeling and is continuing mirdametinib therapy. Following 6 prior revision surgeries that resulted in persistent pseudarthrosis, the addition of mirdametinib to standard surgical approaches in this patient with OFD resulted in the first bone union. Overall, our preclinical results as well as the single patient reported here demonstrate evidence that MEKi monotherapy, together with standard-of-care surgical approaches, can result in healing of persistent pseudarthrosis in patients with OFD RASopathy.

## Discussion

In this report, we engineered an OFD mouse model expressing the *Met*^ΔJMD^ allele to demonstrate the reduced osteogenic potential of periosteal progenitor cells associated with activation of the MAPK pathway. In patients, OFD lesions are restricted to the bone periosteum. Our results implicate the mesenchymal niche–specific expression of *Met* and *Hgf*, encoding the only known ligand for the MET receptor, in the restricted clinical presentation of bony lesions to the periosteum. In vivo, expression of the *Met*^ΔJMD^ allele was associated with cortical long bone defects in mice, as are observed in patients (32), including less structural resistance to torsion and more cortical porosity. These results are consistent with impaired differentiation and mineralization associated with MAPK activation in the bone periosteum, recapitulating the human condition.

OFD is one of multiple somatic skeletal diseases, including NF1 (33, 34), melorheostosis (35), and others, associated with hyperactive RAS (i.e., RASopathies) or downstream MAPK signaling. These conditions are associated with different skeletal presentations, some of which may predispose individuals to pathologic fracture and subsequent pseudarthrosis. Fractures are often treated with rigid fixation and a BMP2-collagen graft to promote bone healing, although clinical and preclinical studies suggest that MAPK hyperactivation inhibits the osteoanabolic effects of BMP2 (36–39). Surprisingly, our results demonstrate that, despite ERK pathway activation, BMP2 was sufficient to rescue osteogenic differentiation of *Met*^ΔJMD^ osteoprogenitor cells in vitro. These results suggest that the therapeutic efficacy of BMP2 differs between skeletal RASopathies despite their mechanistic convergence on hyperactive MAPK signaling. Further studies of these signaling pathways may uncover a mechanistic basis for this divergent osteogenic response to BMP2.

In cancer, *MET*^ΔJMD^ expression leads to an oncogene addiction, which is described as a molecular switch rendering cells dependent on a singular signal transduction pathway, thereby sensitizing tumors to compounds targeting these pathways. In addition to *MET*^ΔJMD^ expression, high copy number amplification of the *MET* gene also leads to oncogene addiction; however, MET inhibitors are only therapeutically effective with *MET* copy number at least 10 or greater (14, 15). These results suggest that the response to MET inhibitors is dependent on the degree to which persistent *MET* expression sustains the activation of downstream signaling pathways and the degree to which cellular dysfunction is dependent on this signaling. It remains possible, as with low-level *MET* amplification, that feedback or other mechanisms may sustain the activation of downstream pathways without inducing an addiction phenotype. Indeed, our results demonstrated, both in mouse and human primary cells, that MET inhibition did not reverse the cellular or molecular consequences of *MET* expression in OFD. Thus, while mutations resulting in *MET*^ΔJMD^ expression are known to cause OFD, an oncogene addiction-like phenotype was not evident in *Met*^ΔJMD^-expressing mouse skeletal progenitor cells or in OFD patient lesion–derived primary cells. These results are consistent with a lack of transformation or increased cancer risk in patients with OFD who carry somatic or inherited *MET* mutations (1, 4).

In contrast to MET inhibition, direct targeting of downstream MEK signaling with the MEKi mirdametinib or selumetinib rescued osteogenic differentiation and mineralization of *Met*^ΔJMD^ mouse osteoprogenitor cells. Likewise, MEKi reversed the molecular signatures inherent in OFD patient lesion–derived primary cells, including from patients with or without detectable *MET* mutations. Both mirdametinib and selumetinib are approved for use in children as young as 2 years of age for the treatment of pNF and may potentially be repurposed to treat conditions associated with hyperactive MAPK signaling, such as OFD. As part of a compassionate use protocol, we demonstrate the successful healing of a persistent pseudarthrosis in a young child with OFD. Following 6 months on treatment, the therapy was well tolerated and resulted in radiographic evidence of union sufficient to transition from rigid external fixation to a cast. On the basis of this result, we hypothesize that postoperative MEKi may support pseudarthrosis healing and that treatment could potentially be discontinued upon satisfactory healing. Future studies will be required to determine appropriate outcomes justifying discontinuation of treatment.

Results from this study demonstrate a nonsurgical therapeutic opportunity for the treatment of OFD in children, opening future potential for similar studies or treatment in patients with other RASopathy-associated skeletal disease. As with OFD, patients with NF1 are at increased risk of pathologic fracture and subsequent pseudarthrosis that often does not heal despite the use of BMP2 and may ultimately require amputation (36). Furthermore, melorheostosis presents with periosteal bone lesions and is caused by activating somatic variants in the gene encoding MEK1 (35). Therefore, our results, together with observations in other RASopathies, converge to implicate MEK inhibition as an effective treatment across a spectrum of somatic skeletal RASopathies.

## Methods

### Sex as a biological variable.

For human studies, primary cells from both male and female patients were used. For mouse studies, both male and female animals were included, and findings are reported for both sexes.

### Generation of Met^ΔJMD^ and Met^Postn^ mice.

*Met*^ΔJMD^ mice were generated using CRISPR/Cas9 reagents at the Transgenic Technology Center of UT Southwestern Medical Center. The sgRNAs were designed to target the intron upstream (GCACTGGGTCAAAGTCTCCT) and downstream (CACCAGACCGACAAATGGTC) of the JMD-encoding exon 15. CRISPR RNA (crRNA) and trans-activating CRISPR RNA (tracrRNA) were annealed and mixed with Cas9 protein nuclease from IDT to form a ribonucleotide protein complex. Cas9 (25 ng/μL) and each sgRNA (25 ng/μL) were microinjected into the pronucleus of fertilized 1-cell eggs isolated from superovulated females C57BL/6J. The eggs were incubated in media containing cytochalasin-B immediately before and during microinjection to improve egg survival. The surviving eggs were transferred into the oviducts of day 0.5 pseudopregnant recipient Institute of Cancer Research (ICR) females (Envigo) to produce founder mice. Founder mice were genotyped by Sanger sequencing, and a mouse harboring deletion of the exon was outcrossed with C57BL/6 mice for at least 3 generations.

*Met*^Postn^ mice were generated by first crossing *Postn*-Cre mice (40) with *Met^fl^* mice (41) (no. 016974, The Jackson Laboratory). The resulting heterozygous mice were intercrossed to produce control (without *Postn*-Cre or Postn-Cre^+^ *Met^+/+^*) and *Met*^Postn^ mice.

All mice were maintained on a C57BL/6 background.

### Cell culture and differentiation.

BMSCs and PEC mesenchymal skeletal progenitor cells were isolated as previously described (42). Hind limb long bones were dissected and liberated of soft tissues. Epiphyses were removed and BMSCs were flushed and expanded in complete α-MEM media (catalog no. 12571-063, Gibco, Thermo Fisher Scientific) supplemented with 10% FBS (catalog no. A56708-01, Thermo Fisher Scientific) and 1% penicillin/streptomycin (PS) (catalog no. 15140-122, Thermo Fisher Scientific). The flushed bones were plated in α-MEM supplemented with 20% FBS and 1% PS, and PECs were allowed to migrate out of the explants. Cells were washed with PBS (catalog no. SH30028.02, Hyclone), passaged using Trypsin/EDTA solution (catalog no. CC-5012, Lonza), and maintained in α-MEM supplemented with 10% FBS and 1% PS. The cells were tested throughout the study to confirm negativity for mycoplasma contamination.

Osteogenic differentiation was achieved by supplementing the complete α-MEM media with 100 μg/mL l-ascorbic acid 2-phosphate (catalog no. A92902, MilliporeSigma) and 5 mM β-glycerophosphate (catalog no. 50020, MilliporeSigma) for 14 days. Media were refreshed every 3 days. RNA was extracted from cultured cells using TRIzol (catalog no. 15596018, Thermo Fisher Scientific). cDNA synthesis was performed using the High Capacity RNA-to-cDNA Kit (catalog no. 4387406, Thermo Fisher Scientific).

For pharmacologic inhibitor experiments, primary mouse cells were treated with vehicle (DMSO, catalog no. D2650, MilliporeSigma), the MET inhibitor capmatinib (10 μM, catalog no. S2788, Selleck Chemicals), the MEK inhibitor mirdametinib (1 μM, catalog no. S1036, Selleck Chemicals), or the MEK inhibitor selumetinib (5 μM, catalog no. S1008, Selleck Chemicals). For time-course experiments, mouse cells were serum starved overnight and treated with 1.5 nM HGF (catalog no. H9661, MilliporeSigma) for 5 minutes, and then media were replaced with serum-free α-MEM for the indicated durations.

Gene expression of undifferentiated and differentiated samples was evaluated using SYBR Green PCR Master Mix (catalog no. 4364344, Thermo Fisher Scientific). Primer sequences can be found in Supplemental Table 8. Target specificity was evaluated by melting curve analysis.

### Cell staining.

Following osteogenic differentiation, the media were removed, and the cells were washed once with PBS followed by cell fixation in 50% ethanol for 15 minutes at 4°C and incubation with 1% (wt/vol with 0.1% ammonium hydroxide) alizarin red S (catalog no. A5533, MilliporeSigma). The staining was then washed 3 times with water, dried, and imaged. For quantification, alizarin red stain was eluted with 10% cetylpyridinium monohydrate chloride (catalog no. 190177, MP Biomedical), and OD was measured at 570 nm. For crystal violet staining, cells were washed once in PBS at room temperature and incubated with 2% (wt/vol with 0.2% ethanol) crystal violet (catalog no. C0775, MilliporeSigma) at room temperature. The staining was washed with water, dried, and imaged. For quantification, crystal violet stain was eluted with methanol, and OD was measured at 570 nm.

### Cell-cycle analysis.

Trypsinized cell suspensions were centrifuged at 300*g* for 5 minutes, washed in PBS, and fixed in cold ethanol for 2 hours. Following fixation, cells were pelleted, washed with PBS, and resuspended in propidium iodide (PI) with RNase staining solution (catalog no. ab139418, Abcam) at 37°C for 30 minutes in the dark. Data were acquired using a BD FACSAria II and analyzed using BD FACSDiva 9.0.1

### Histology and quantification.

Femurs were fixed in 4% paraformaldehyde overnight and maintained in PBS. Decalcification in 14% EDTA (CAS no. 6381-92-6, MilliporeSigma) and paraffin embedment were performed at the UTSW Histopathology core facility using a Thermo Excelsior Tissue Processor and Sakura TEC6 Embedding Center. Samples were processed with vacuum assist beginning in 70% ethanol followed by 6-graded exchanges of ethanol, 3 exchanges of xylene, and 3 exchanges of molten paraffin. Five-micron sections were cut on a Leica RM2255 rotary microtome. The presence of osteoclasts was confirmed by tartrate-resistant acid phosphatase (TRAP) staining. In brief, slides were deparaffinized and rehydrated through multiple xylenes, 100% ethanol, and 95% ethanol, ending in deionized water. To facilitate enzyme activity, slides were incubated in a heated solution containing 0.01% napthol AS-BI phosphate substrate and basic stock incubation medium. After incubation, slides were directly transferred to a heated solution containing basic stock incubation medium and pararosaniline dye to visualize precipitates from the enzyme activity. After sufficient staining, dye development was halted through three 5-minute changes of deionized water. Following rinses, sections were then counterstained in a 0.02% Fast Green solution (MilliporeSigma). Excess counterstain was removed by a quick change of deionized water. Tissue sections were then dehydrated and cleared before coverslips were applied with synthetic mounting media.

Paraffin sections for IHC were cut at 4 μm thickness, mounted onto slides, and dried overnight at 42°C. Selected slides were incubated in 60°C for 10 minutes, deparaffinized in xylene, rehydrated through a descending grade of alcohol (100%, 95%, 70%), and then washed in distilled water. Endogenous peroxidase was deactivated with 3% H_2_O_2_ in methanol at room temperature and rinsed with distilled water and transferred to PBS with 0.05% Tween-20 (PBST), pH 7.4 (P3563-10PAK, MilliporeSigma). Sections were blocked with 10% normal goat serum (catalog no. ab7481, Abcam) for 1 hour at room temperature to reduce nonspecific binding and incubated overnight at 4°C with rabbit polyclonal anti-Sp7/anti-osterix antibody (catalog no. ab22552, Abcam) at a dilution of 1:10,000. Sections were washed 3 times with PBST and incubated with secondary goat, anti–rabbit IgG HRP–conjugated antibody (AP187P, MilliporeSigma) at 1:500 dilution for 1 hour 30 minutes and washed 3 times in PBST. The chromogen DAB solution (catalog no. ab64238, Abcam) was placed in sections for 10 minutes to visualize brown reaction and then washed in distilled water. Sections were counterstained with Mayer’s hematoxylin (MHS80-2.5L, MilliporeSigma) for 2 minutes and washed in running water, dehydrated in an ascending grade of alcohol (70%, 95%, 100%), cleared in xylene, and then mounted in Cytoseal XYL (catalog no. 8312-4, Thermo Fisher Scientific). Negative controls were run in parallel by replacing the primary antibody with blocking solution.

Slides were scanned using a Motic EasyScan, with quantification performed using Motic Digital Slide Assistant software (version 1.0.7.61b).

### Patient primary cell culture.

Patient-derived primary cells were provided by the Scottish Rite for Children Biorepository. Briefly, patient-derived skeletal specimens were finely chopped and digested in 0.25 mg/mL collagenase I (catalog no. 17100017, Thermo Fisher Scientific) and 1 mg/mL dispase (catalog no. 17105041, Thermo Fisher Scientific) in DMEM (catalog no. 11965092, Thermo Fisher Scientific) supplemented with 15% FBS and 1% PS overnight at 37°C. Undigested tissue was removed by centrifugation, and the cells were resuspended and plated in α-MEM supplemented with 20% FBS and 1% PS. Primary cells were maintained in α-MEM supplemented with 10% FBS and 1% PS until confluence was reached. Cells were then washed with PBS and passaged using Trypsin/EDTA solution (catalog no. CC-5012, Lonza). The cells were tested for mycoplasma contamination throughout the study. All experiments were performed with early-passage (passage <4) cells.

For pharmacologic inhibitor experiments, confluent primary cells were treated with vehicle (DMSO, catalog no. D2650, MilliporeSigma), the MET inhibitor capmatinib (10 μM, catalog no. S2788, Selleck Chemicals), the MEK inhibitor mirdametinib (100 nM, catalog no. S1036, Selleck Chemicals), or the MEK inhibitor selumetinib (5 μM, catalog no. S1008, Selleck Chemicals) for 24 hours.

### MET splicing and variant detection.

Total RNA was extracted from cultured human primary cells using the RNeasy Plus Mini kit (catalog no. 74134, Qiagen). Cells were washed twice with PBS, followed by harvesting with RLT plus buffer from the kit supplemented with β-mercaptoethanol (catalog no. M-7522, MilliporeSigma). The extraction was performed according to the manufacture’s recommendations. RNA was eluted into 30 μL RNase-free water. cDNA was synthesized by RT using the High-Capacity RNA-to-cDNA kit (catalog no. 4387406, Thermo Fisher Scientific). Next, RT-PCR was performed to amplify the *MET* transcript, and amplicons were resolved by agarose gel electrophoresis. Separated amplicons were excised and extracted from the agarose gel using the QIAquick gel extraction kit (catalog no. 28704, Qiagen) following the manufacturer’s recommendations. Sanger sequencing was performed to identify individual amplicon splice variants.

DNA was extracted from whole blood or lesion-derived cultured primary cells using the QIAmp DNA extraction kit (catalog no. 51304, Qiagen) according to the manufacturer’s recommendations. The DNA was used to amplify *MET* exon 14. The amplicons were run on agarose gel to check the correct product size. The amplicons were cleaned up using ExoSAP-IT (catalog no. 78200200UL, Thermo Fisher Scientific) prior to sequencing. Amplicons were then Sanger sequenced to identify potential somatic mutations. Primer sequences are listed in Supplemental Table 8. The Sanger sequencing data were analyzed using Sequencher, version 5.1 (Gene Codes Corporation).

### ddPCR.

DNA extracted from blood samples or from primary cells cultured from the lesion bone were analyzed by ddPCR. The assay was ordered using Bio-Rad’s online tool for Custom ddPCR assays (Bio-Rad Laboratories) (assay ID: dHsaMDS847883628). A master mix containing the primers (900 nM), probes (250 nM) for the mutant allele (labeled with FAM) and reference allele (labeled with HEX), the DNA template (200 ng), and 2× ready-to-use ddPCR Supermix (no dUTP) were prepared in a 22 μL volume according to the manufacturer’s guidelines (Bio-Rad). We also added 1 μL HaeIII enzyme to each reaction to improve template accessibility via fragmentation of genomic DNA that is randomly subdivided into droplets that undergo PCR independently.

Droplets generated from the reaction mixture using a QX200 Auto Droplet Generator were amplified using the following PCR cycling protocol: a 95°C enzyme activation step for 5 minutes followed by 40 cycles of a 2-step cycling protocol (95°C for 30 seconds and 58°C for 1 minute) and a final denaturation step at 98°C for 10 minutes, in accordance with the manufacturer’s instructions (Bio-Rad). The amplification signal was measured using a QX200 Droplet Reader, and data analysis was performed using QuantaSoft software, version 1.7.4 (Bio-Rad).

### Transcriptome sequencing.

Total RNA was extracted from cultured patient-derived primary cells using the RNeasy Plus Mini kit (catalog 74134, Quiagen). The quality of RNAs was evaluated using an Agilent TapeStation 4200 (Agilent Technologies). Transcriptome sequencing libraries were prepared using the TruSeq Stranded Total RNA LT sample preparation kit to deplete ribosomal RNA prior to cDNA synthesis. Afterwards, indexed adaptors were ligated to cDNA fragments and purified using AMPureXP beads (Beckman Coulter Life Sciences). Sequencing libraries were normalized prior to sequencing using the 150 bp paired-end protocol on an Illumina NextSeq 2000 instrument.

Transcriptome analyses were conducted using Partek Flow software. Sequence reads were mapped to the human reference genome (hg38) using STAR (version 2.7.8). Alignments were filtered to remove low-quality reads prior to quantification of gene expression using the Ensembl transcript database. Gene expression was normalized using the median ratio method for DESeq2 analysis. For principal component analysis, systematic differences between patients were removed using general linear modeling. Differential gene expression analysis was performed using DESeq2 and included patient and treatment variables. Differentially expressed genes were defined as at least a 2-fold change in expression and a FDR *P* value of less than 0.05. Differential gene expression analysis, principal component analysis, heatmap plotting, and hierarchical clustering were performed using Partek Flow software (Illumina).

### Western blotting.

Protein was extracted using RIPA buffer (catalog no. 89900, Thermo Fisher Scientific) with a halt protease inhibitor cocktail (catalog no. 78430, Thermo Fisher Scientific) and phosphatase inhibitor cocktails 2 and 3 (catalog no. P5726 and P0044, MilliporeSigma). Concentration was determined using the Pierce BCA Protein Assay kit (catalog no. 23227, Thermo Fisher Scientific). Lysate samples were denatured in the sample buffer (catalog no. BP111NR, Boston Bioproducts) supplemented with β-mercaptoethanol. Equal amounts of sample (in micrograms) were resolved in 10% polyacrylamide gels (catalog no. M00664 and M00666, GenScript) for 1 hour 15 minutes at 120 volts. The wet transfer of resolved proteins was performed for 1 hour at 100 volts at 4°C. Antibody detection was performed using rabbit anti–total ERK (anti–t-ERK) (1:1,000; catalog no. 4695S, Cell Signaling Technology); rabbit anti–p-ERK (1:1,000; catalog no. 4370S, Cell Signaling Technology); rabbit anti-S6 (1:1,000; catalog no. 4858, Cell Signaling Technology); and rabbit anti–p-S6 (1:1,000; catalog no. 4858, Cell Signaling Technology) primary antibodies in 1× TBST buffer (10× TBS, catalog no. 1706435, Bio-Rad; Tween-20, catalog no. P9416, MilliporeSigma) with 5% BSA (catalog no. A7906, MilliporeSigma) overnight at 4°C. After washing 3 times with 1× TBST buffer, goat anti-rabbit (1:5,000) IRDye secondary antibodies (catalog no. 926-32211, LI-COR Biosciences) were incubated in 5% nonfat dry milk in 1× TBST buffer for 1 hour at room temperature in the dark. Following 2 washes with 1× TBST buffer and 1 wash with 1× TBS buffer, images were acquired using the Odyssey CLx system (LI-COR Biosciences). Western quantification was performed using ImageJ software (NIH) for human samples and the LI-COR Odyssey machine for mouse samples.

### Micro-CT.

Femurs were harvested from 4-month-old control and *Met*^ΔJMD^ mice and imaged using a Skyscan 1072 X-ray Microtomography (Skyscan, software version 1.5) set with a voltage of 50 kV, a current of 201 μA, an exposure time of 650 ms, a 0.5 mm Al filter, an average frame of 6, and a rotation step of 0.4° per projection. A scout view of each bone was taken, and the sample height was adjusted to ensure the bone was within the field of view. Solid 3D models were reconstructed using NRecon (Skyscan, version 1.7.4.6), giving a resolution of 8 μm, and the trabecular parameters were measured using methods recommended by Skyscan. Regions of interest were analyzed using CTAn software (version 1.20.3.0). The trabecular parameters were calculated on 200 slices of trabecular bone from 50 slices below the growth plate, with a threshold of 90–255 grayscale values. Measurements were calculated using the American Society of Bone and Mineral Research nomenclature (43). Likewise, the cortical parameters were calculated on 100 slices from mid-diaphysis, with a threshold of 138–255 grayscale values.

### Biomechanical testing.

Hydrated, intact femurs were loaded to failure in 3-point bending at a rate of 3 mm/min using a material testing instrument (Instron Dynamight 8841, Instron Inc.) to assess the biomechanical properties of the cortical bone. Each femur was centered on the 3-point bending fixture with the medial side facing forward and the anterior side facing down (i.e., anterior in tension). The span between the 2 supports was 8 mm for all tests. The linear variable differential transformer (LVDT) attached to the linear actuator and a 100 N load cell mounted in line with the actuator recorded the force versus displacement data at a sampling rate of 50 Hz. As described previously (44, 45), these data were analyzed to determine stiffness.

### Statistics.

Except for transcriptome analyses, all data are represented as the mean and SEM from multiple replicates from independent experiments performed on different days. Most data were normalized using log transformation, and including 2-sided *t*-test, 1-way ANOVA, and 2-way ANOVA were performed using GraphPad Prism (GraphPad Software). Sample sizes were not predetermined, and mice were randomly assigned to experiments. Sex-matched littermate controls were used for all experiments. A *P* value of less than 0.05 was considered significant.

### Study approvals.

Patient-derived primary cells were provided by the Scottish Rite for Children Biorepository following written informed consent and approved by the IRB at UT Southwestern Medical Center (STU no. 092011-034) for the collection and use of discarded surgical waste, including excess graft and resected pseudarthrosis tissue. Off-label compassionate use treatment with mirdametinib was administered following FDA and UT Southwestern Medical Center IRB regulatory approval and parental informed consent (IND no. 175099). Mirdametinib was provided by SpringWorks Therapeutics Inc. (Stamford, Connecticut, USA) as part of its compassionate use program. All rodent procedures were approved by the IACUC of UT Southwestern Medical Center (APN no. 2016-101455).

### Data availability.

Supporting data values are provided in the Supporting Data Values file. RNA-seq data are available in the NCBI’s Gene Expression Omnibus (GEO) database (GEO GSE305253).

## Author contributions

ABK, CAW, and JJR designed the research study. ABK, KD, NP, IO, MW, RC, SU, GJJ, CRF, REH, JR, MN, DAP, and LJK conducted experiments. ABK, NP, IO, SU, JSN, GJJ, CRF, and JJR analyzed data. SJC provided reagents. AK and JJR wrote the manuscript. All authors reviewed and edited the manuscript.

## Conflict of interest

The authors have declared that no conflict of interest exists.

## Funding support

This work is the result of NIH funding, in whole or in part, and is subject to the NIH Public Access Policy. Through acceptance of this federal funding, the NIH has been given a right to make the work publicly available in PubMed Central.

- Pediatric Orthopaedic Society of North America award no. 822001 (to JJR).
- Department of Defense award W81XWH-22-1-0576 (to JJR).
- Scottish Rite for Children (to JJR).
- NIH grant ZIA HD009024 (to CRF).

## Supplementary Material

Supplemental data

Unedited blot and gel images

Supplemental tables 1-7

Supplemental table 8

Supporting data values

## Version 1 02/26/2026

In-Press Preview

## Version 2 05/01/2026

Electronic publication
