# Supplementary material for: MEK inhibitor mirdametinib promotes fracture healing in osteofibrous dysplasia RASopathy
Source: J Clin Invest. 2026 Feb 26;136(9):e199048. doi: 10.1172/JCI199048 (PMC13132394; doi:10.1172/JCI199048)
Supplement: Supplemental data [file jci-136-199048-s096.pdf]

Supplemental Figure 1

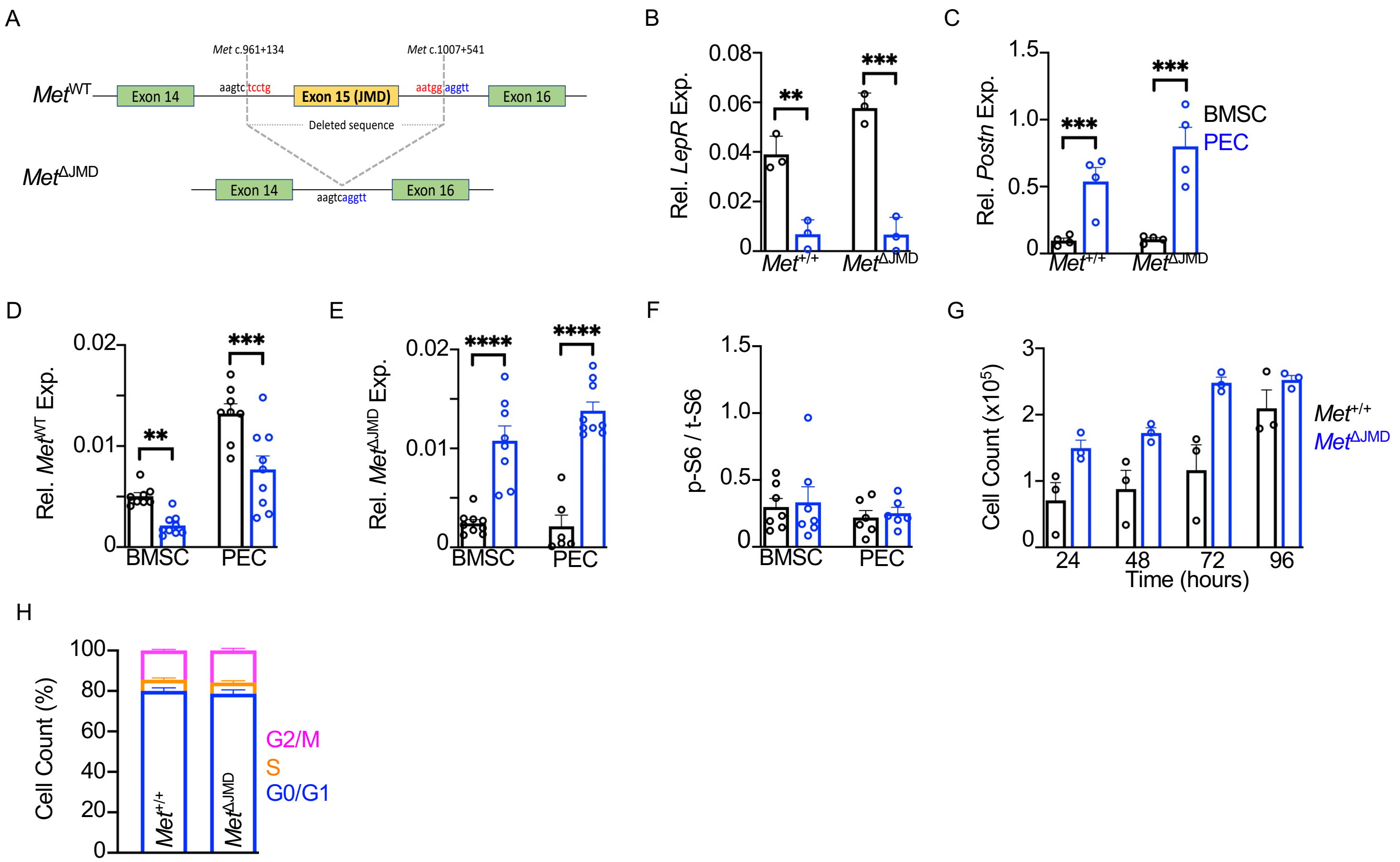

Supplemental Figure 2

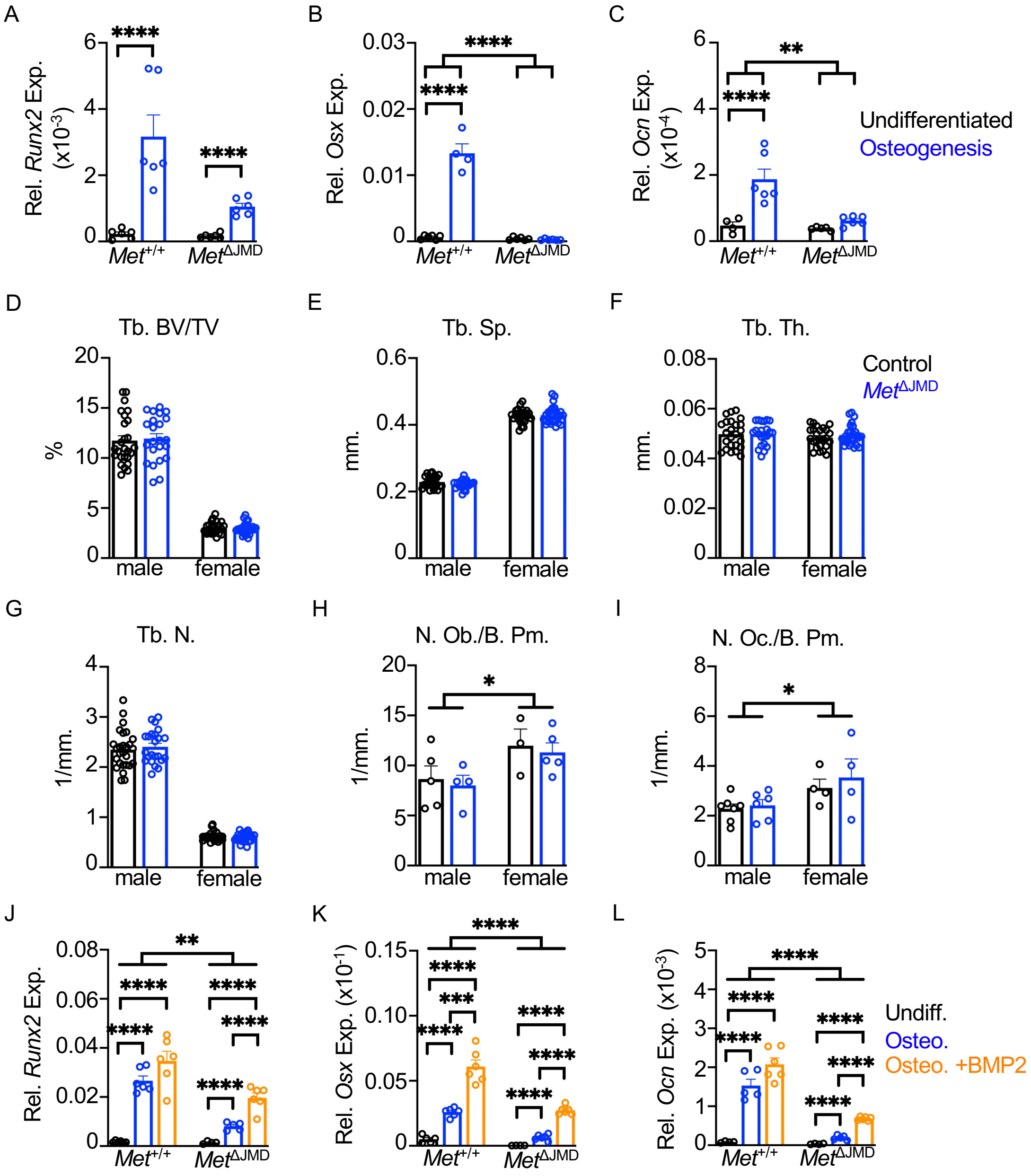

Supplemental Figure 3

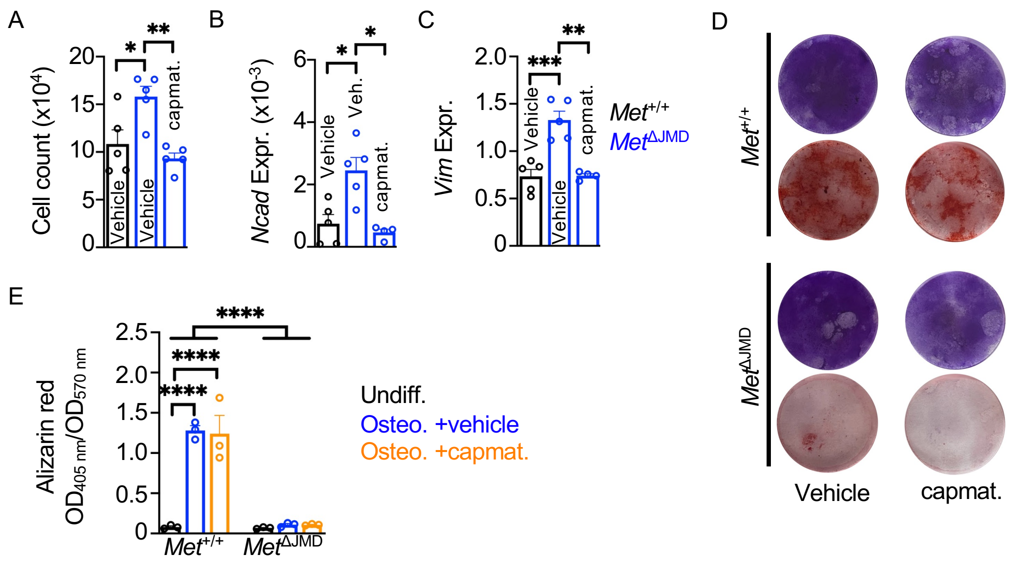

Supplemental Figure 4

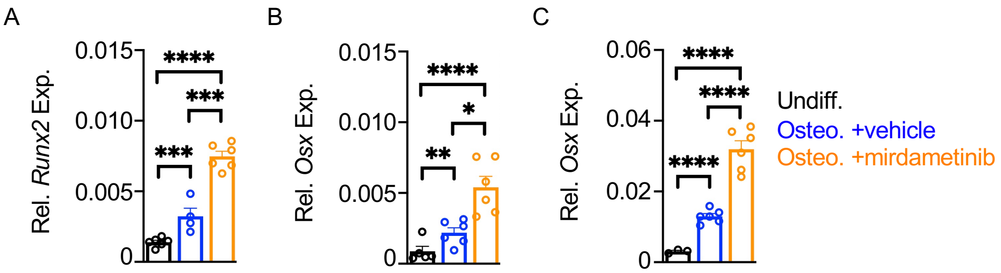

Supplemental Figure 5

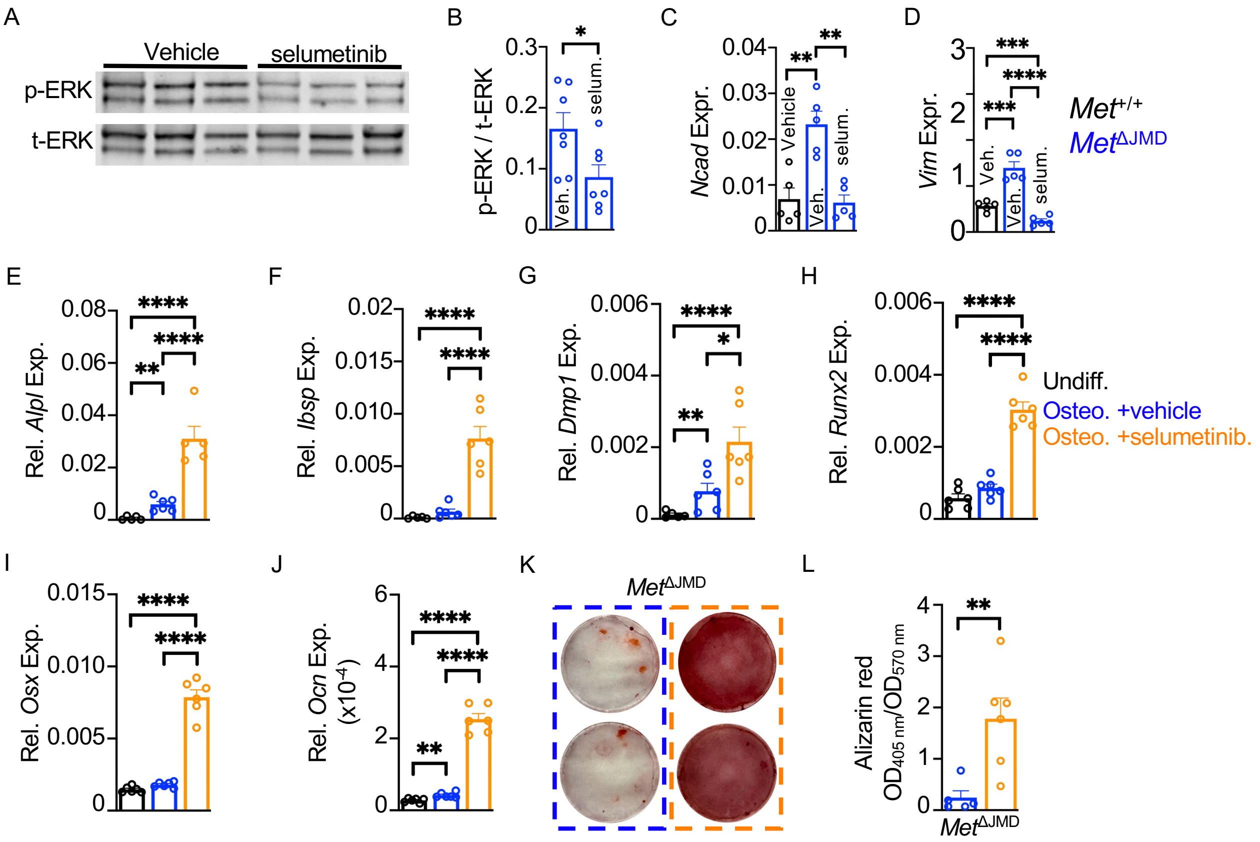

Supplemental Figure 6

A

B

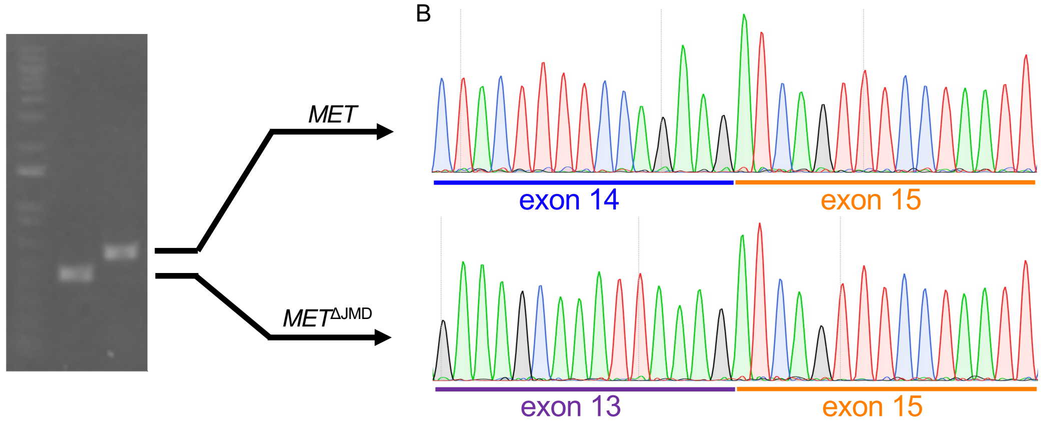

C

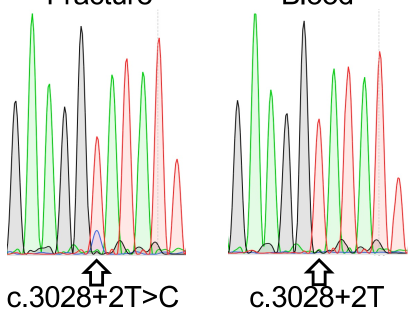

D

	Replicate 1			Replicate 2		
	Mutant allele (copies/µL)	Ref. allele (copies/µl)	VAF (%)	Mutant allele (copies/µL)	Ref. allele (copies/µl)	VAF (%)
Lesion	266	1,474	18.1	229	1,131	20.2
Blood	0	2,883	0	0	2,768	0

Supplemental Figure 7

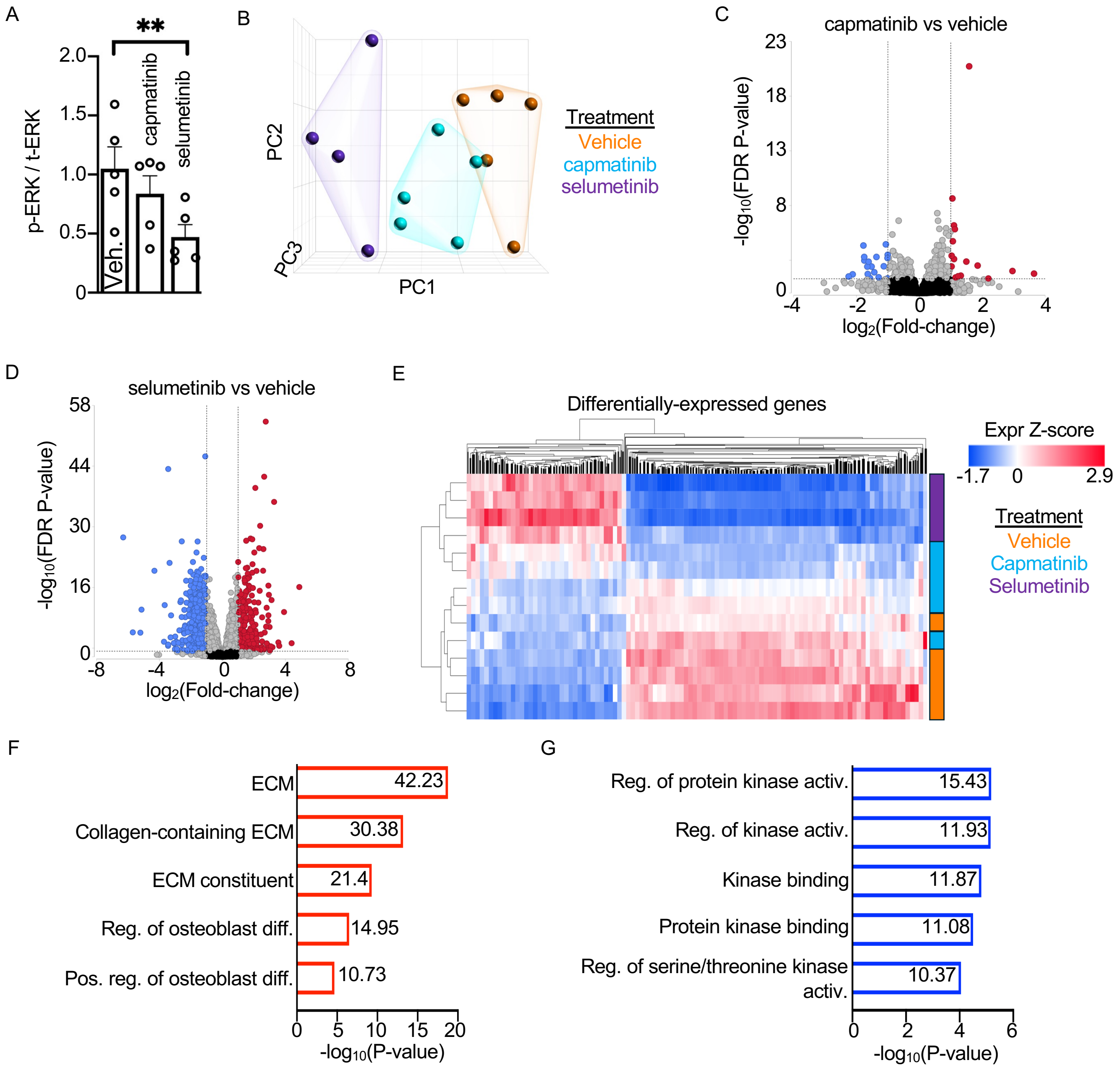

Supplemental Figure 1. Confirmation of skeletal progenitor cell populations. **(A)** Schematic of the *Met* locus indicating the location of the exon 15 deletion in $Met^{\Delta JMD}$ mice. **(**B and **C)** Relative expression of the BMSC marker gene *LepR* (B) and the PEC marker gene *Postn* (C) in control ($Met^{+/+}$; n=3-4) and $Met^{\Delta JMD}$ (n=3-4) BMSCs (black) and PECs (blue). Statistically significant differences were determined by 2-way ANOVA with Sidak multiple test correction. **(D** and **E)** Allele-specific expression of wild-type (D) and mutant (E) *Met* alleles in control ($Met^{+/+}$; n=6-9) and $Met^{\Delta JMD}$ (n=8-9) BMSCs and PECs. Statistically significant differences were determined by 2-way ANOVA with Sidak multiple test correction. **(F)** Western blot quantification of S6 activation in serum-starved BMSCs and PECs from control (n=7) and $Met^{\Delta JMD}$ (n=6) mice. **(G)** Time-course cell count quantification of PECs from control (n=3) and $Met^{\Delta JMD}$ (n=3) mice. Statistically significant differences were determined by 2-way ANOVA with Sidak multiple test correction. **(H)** Quantification of cell cycle stage by flow cytometric analysis of PECs from control and $Met^{\Delta JMD}$ mice (n=3 per group). RT-qPCR from cultured cells and other quantification data are presented as mean ± SEM. **, p<0.01; ***, p<0.001; ****, p<0.0001.

Supplemental Figure 2. Microstructure analysis of adult $Met^{\Delta JMD}$ mice. **(A-C)** Relative expression of the osteogenic genes *Runx2* (A), *Osx* (B), and *Ocn* (C) in control (n=4-6) and $Met^{\Delta JMD}$ (n=6) PECs prior to (undifferentiated, black) and following osteogenic differentiation (Osteogenesis, blue). Statistically significant differences were determined by 2-way ANOVA with Sidak multiple test correction. **(D-G)** Distal femur trabecular bone volume/tissue volume (D; Tb. BVTV), trabecular spacing (E; Tb. Sp.), trabecular thickness (F; Tb. Th.), and trabecular number (G; Tb. N.) in control (black; n=49-52) and $Met^{\Delta JMD}$ (blue; n=50-51) male and female mice. Statistically significant differences were determined by 2-way ANOVA with Sidak

multiple test correction. **(H-I)** Osteoblast number per trabecular bone perimeter (H; N. Ob./B. Pm.) and osteoclast number per trabecular bone perimeter (I; N. Oc./B. Pm.) in control (black; n=3-7) and *Met*$^{\Delta JMD}$ (blue; n=4-6) male and female mice. Statistically significant differences were determined by 2-way ANOVA with Sidak multiple test correction. **(J-L)** Relative expression of the osteogenic genes *Runx2* (J), *Osx* (K), and *Ocn* (L) in control (n=4-6) and *Met*$^{\Delta JMD}$ (n=4-6) PECs prior to (undifferentiated, black) or following standard osteogenic differentiation (Osteo., blue) or osteogenic differentiation with BMP2 (Osteo.+BMP2, orange). Statistically significant differences were determined by 2-way ANOVA with Tukey multiple test correction. RT-qPCR from cultured cells and other quantification data are presented as mean ± SEM. *, p<0.05; **, p<0.01; ***, p<0.001; ****, p<0.0001.

Supplemental Figure 3. Response of *Met*$^{\Delta JMD}$ PECs to capmatinib treatment. **(A)** Quantification of cell adhesion in control (*Met*$^{+/+}$) or *Met*$^{\Delta JMD}$ (blue) PECs treated with vehicle (Veh.) or capmatinib (capmat.) (n=5 per group). Statistically significant differences were determined by 1-way ANOVA with Tukey multiple test correction. **(B** and **C)** Relative expression of cell adhesion genes *Ncad* (B) and *Vim* (C) in control or *Met*$^{\Delta JMD}$ PECs treated with vehicle or capmatinib (n=4-5 per group). Statistically significant differences were determined by 1-way ANOVA with Tukey multiple test correction. **(D)** Representative Alizarin red and Crystal Violoet (CV) staining following osteogenic differentiation of control and *Met*$^{\Delta JMD}$ PECs treated with vehicle or capmatinib. **(E)** Quantification of alizarin staining of control (*Met*$^{+/+}$) and *Met*$^{\Delta JMD}$ PECs prior to (Undiff.) or following osteogenic differentiation (Osteo.) with vehicle or capmatinib (capmat.) (n=3 per group). Statistically significant differences were determined by 2-way ANOVA with Tukey multiple test correction. RT-qPCR from cultured cells and other

quantification data are presented as mean ± SEM. *, p<0.05; **, p<0.01; ***, p<0.001; ****, p<0.0001.

Supplemental Figure 4. Response of *Met*$^{\Delta JMD}$ PECs to mirdametinib treatment. **(A-C)** Relative expression of the osteogenic genes *Runx2* (A), *Osx* (B), and *Ocn* (C) in *Met*$^{\Delta JMD}$ PECs prior to (Undiff., black) or following osteogenic differentiation in the presence of vehicle (Osteo.+vehicle, blue) or mirdametinib (Osteo.+ mirdametinib, orange) (n=3-6 per group). Statistically significant differences were determined by 1-way ANOVA with Tukey multiple test correction. RT-qPCR from cultured cells and other quantification data are presented as mean ± SEM. *, p<0.05; **, p<0.01; ***, p<0.001; ****, p<0.0001.

Supplemental Figure 5. Response of *Met*$^{\Delta JMD}$ PECs to selumetinib treatment. **(A** and **B)** Representative western blot (A) and quantification (B) demonstrating reduced ERK pathway activation with selumetinib treatment (n=7 per group). Statistically significant differences were determined by T-test. **(C** and **D)** Relative expression of cell adhesion genes *Ncad* (C) and *Vim* (D) in control (*Met*$^{+/+}$) or *Met*$^{\Delta JMD}$ PECs treated with vehicle or selumetinib (n=5 per group). Statistically significant differences were determined by 1-way ANOVA with Tukey multiple test correction. **(E-J)** Relative expression of the osteogenic genes *Alpl* (E), *Ibsp* (F), *Dmp1* (G), *Runx2* (H), *Osx* (I), and *Ocn* (J) in *Met*$^{\Delta JMD}$ PECs prior to (Undiff., black) or following osteogenic differentiation in the presence of vehicle (Osteo.+vehicle, blue) or selumetinib (Osteo.+selumetinib, orange) (n=5-6 per group). Statistically significant differences were determined by 1-way ANOVA with Tukey multiple test correction. **(K)** Representative Alizarin red staining following osteogenic differentiation of *Met*$^{\Delta JMD}$ PECs treated with vehicle (blue) or

selumetinib (orange). **(L)** Quantification of alizarin staining *Met*$^{\Delta JMD}$ PECs following osteogenic differentiation with vehicle (blue; n=5) or selumetinib (orange; n=6). Statistically significant differences were determined by T-test. RT-qPCR from cultured cells and other quantification data are presented as mean ± SEM. *, p<0.05; **, p<0.01; ***, p<0.001; ****, p<0.0001.

Supplemental Figure 6. Somatic *MET* mutation in human sporadic OFD. **(A** and **B)** RT-PCR (A) and Sanger sequencing (B) confirmation of *MET*$^{\Delta JMD}$ expression in clonal amplicons from pseudarthrosis-derived primary cells from an OFD patient. **(C** and **D)** Sanger sequence confirmation (C) and droplet digital PCR quantification (D) of the somatic splice-site mutation c.3028+2T>C using DNA from cultured pseudarthrosis-derived primary cells or patient-matched blood from an OFD patient.

Supplemental Figure 7. Molecular characterization of patient OFD-derived primary cells. **(A)** Quantification of ERK pathway activation in OFD lesion-derived primary cells treated with vehicle (Veh.), capmatinib, or selumetinib (n=5 per group). Quantification is presented as mean ± SEM. with significant differences determined by 1-way ANOVA with Dunnett multiple test correction. **(B)** Principal component analysis from RNA-seq analysis of OFD pseudarthrosis-derived primary cells treated with vehicle (orange), capmatinib (blue), or selumetinib (purple). **(C** and **D)** Volcano plot showing differentially expressed genes between capmatinib (C) or selumetinib (D) treated samples compared to vehicle. **(E)** Heatmap of genes differentially expressed following selumetinib treatment across all samples treated with vehicle (orange), capmatinib (blue), or selumetinib (purple). **(F** and **G)** Gene ontology analysis of differentially

expressed genes with increased (F) or decreased (G) expression following selumetinib treatment. Enrichment values for each category are indicated. **, $p<0.01$.
