# Supplementary material for: MEK inhibitor mirdametinib promotes fracture healing in osteofibrous dysplasia RASopathy
Source: J Clin Invest. 2026 Feb 26;136(9):e199048. doi: 10.1172/JCI199048 (PMC13132394; doi:10.1172/JCI199048)
Supplement: Unedited blot and gel images [file jci-136-199048-s097.pdf]

Full unedited blot/gel for Figure 1A

Blot1

Primary Ab:
Rabbit tErk [cell signaling # 4695S (1:1000] in 5% BSA in TBST

Secondary Ab:
Goat anti-rabbit (1:5000) in 5% milk TBST

Blot2

Primary Ab:
Rabbit pErk [cell signaling # 4370S (1:1000)] in 5% BSA in TBST

Secondary Ab:
Goat anti-rabbit (1:5000) in 5% milk TBST

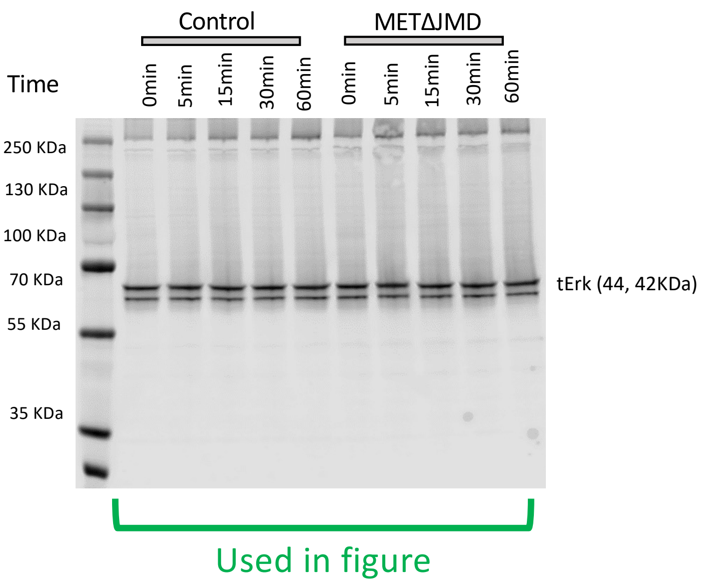

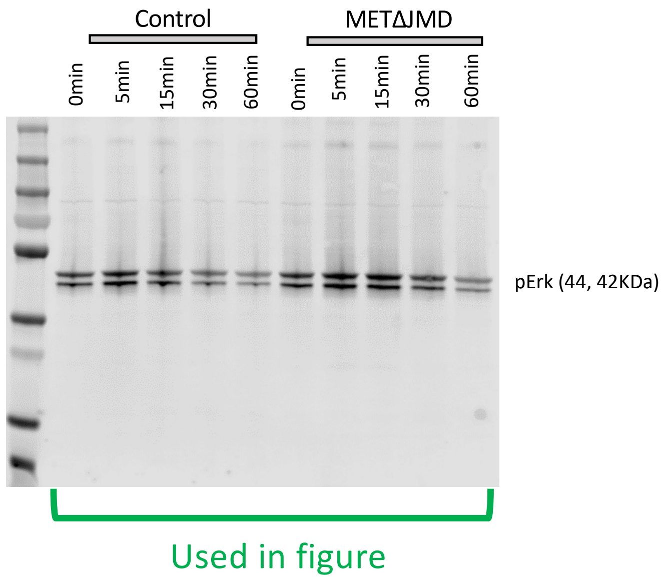

Expected product size:
pErk/tErk: 44, 42KDa

Full unedited blot/gel for Figure 1E

15ug protein loaded

Blot1

Primary Ab:
Rabbit tERK [cell signaling # 4695S (1:1000] in 5% BSA in TBST

Secondary Ab:
Goat anti-rabbit (1:5000) in 5% milk TBST

Blot2

Primary Ab:
Rabbit pERK [cell signaling # 4370S(1:1000)] in 5% BSA in TBST

Secondary Ab:
Goat anti-rabbit (1:5000) in 5% milk TBST

Expected product size:
pErk/tErk: 44, 42KDa

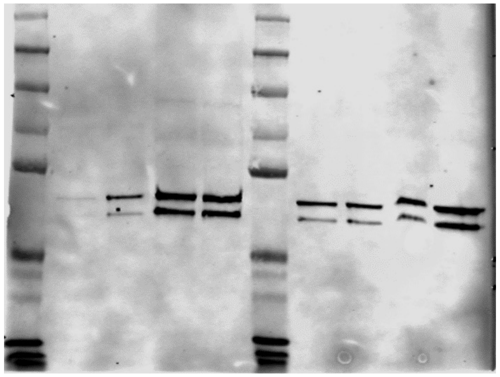

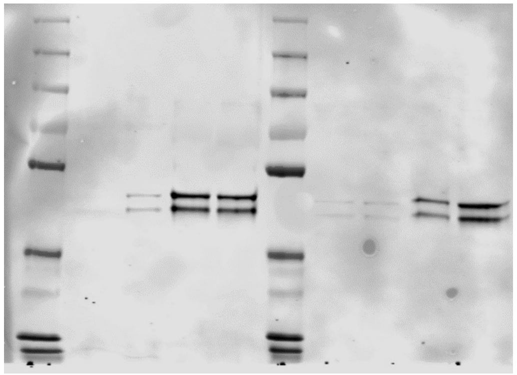

Full unedited blot/gel for Figure 4B

15ug protein loaded

Blot1

Primary Ab:
Rabbit tERK [cell signaling # 4695S (1:1000] in 5% BSA in TBST

Secondary Ab:
Goat anti-rabbit (1:5000) in 5% milk TBST

Blot2

Primary Ab:
Rabbit pERK [cell signaling # 4370S (1:1000)] in 5% BSA in TBST

Secondary Ab:
Goat anti-rabbit (1:5000) in 5% milk TBST

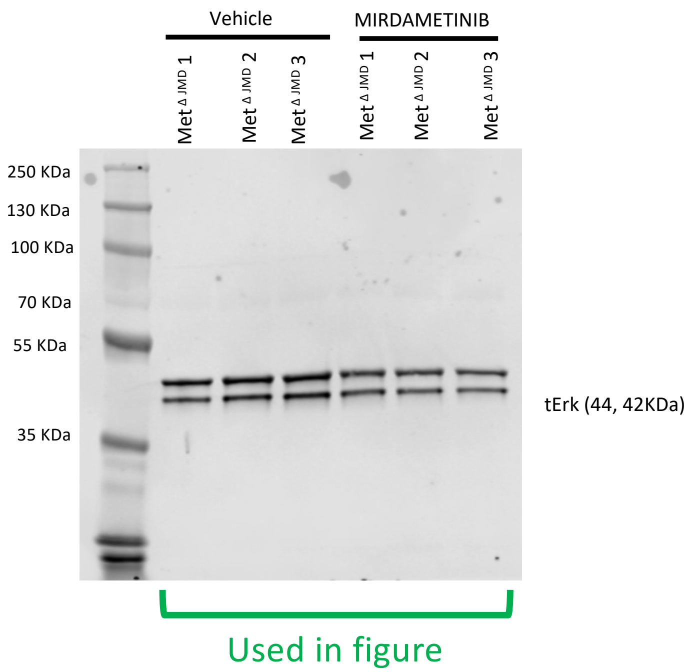

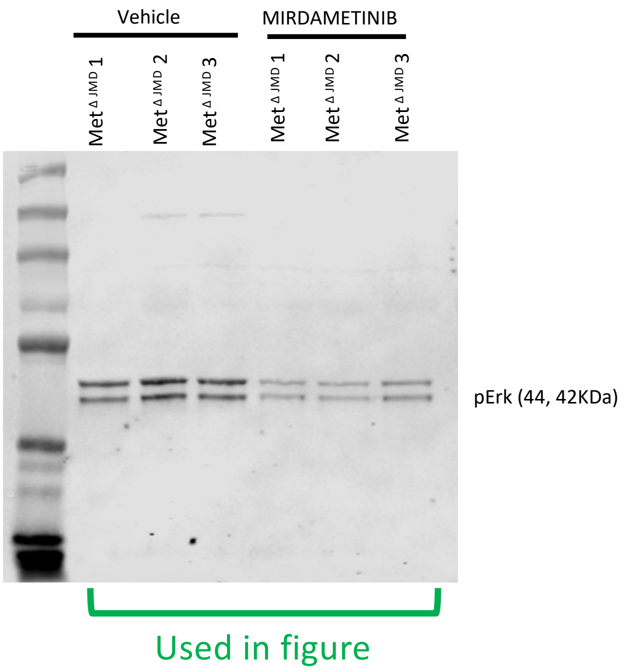

Expected product size:
pErk/tErk: 44, 42KDa

Full unedited blot/gel for Figure 5A

8 ug protein loaded

Blot1
Primary Ab:
Rabbit pERK [cell signaling # 4370S (1:1000] in 5% BSA in TBST

Secondary Ab:
Goat anti-rabbit (1:10,000) in 5% milk TBST

Blot2
Primary Ab:
Rabbit tERK [cell signaling # 4695S (1:2000)] in 5% BSA in TBST

Secondary Ab:
Goat anti-rabbit (1:10,000) in 5% milk TBST

Expected product size:
pERK/tERK: 42/44 kDa

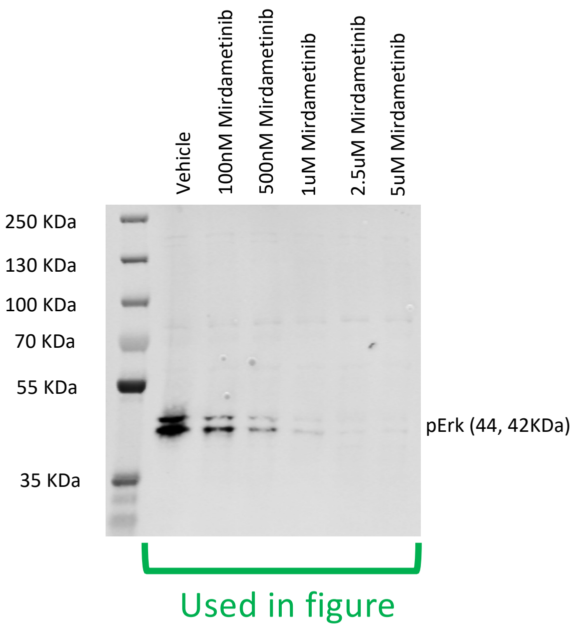

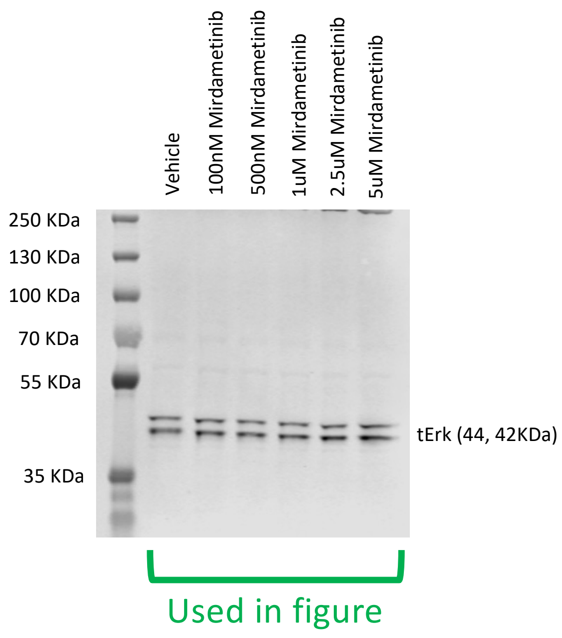

Full unedited blot/gel for Figure S5A

15ug protein loaded

Blot1

Primary Ab:
Rabbit tERK cell signaling # 4695S(1:1000) in 5% BSA in TBST

Secondary Ab:
Goat anti-Rabbit (1:5000) in 5% milk TBST

Blot2

Primary Ab:
Rabbit pERK [cell signaling # 4370S (1:1000)] in 5% BSA in TBST

Secondary Ab:
Goat anti-rabbit (1:5000) in 5% milk TBST

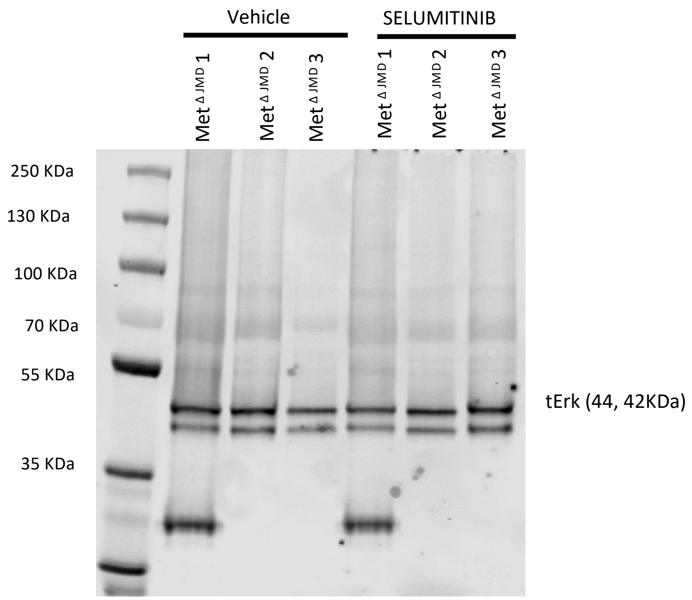

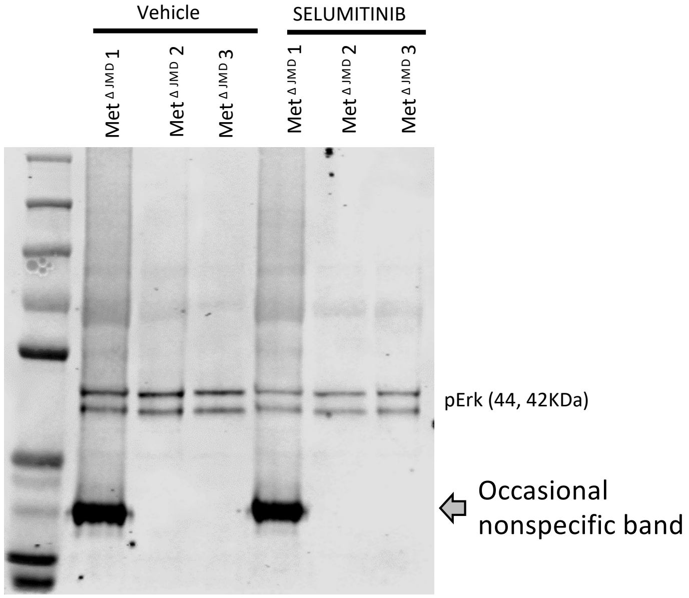

Expected product size:
pErk/tErk: 44, 42KDa
